# Complex II assembly drives metabolic adaptation to OXPHOS dysfunction

**DOI:** 10.1126/sciadv.adr6012

**Published:** 2025-08-15

**Authors:** Roopasingam Kugapreethan, Sheik Nadeem Elahee Doomun, Joanna Sacharz, Ann E. Frazier, Tanavi Sharma, Yau Chung Low, Shuai Nie, Michael G. Leeming, Linden Muellner-Wong, Karena Last, Tegan Stait, David P. De Souza, David R. Thorburn, Malcolm J. McConville, David A. Stroud

**Affiliations:** ^1^ Department of Biochemistry and Pharmacology, Bio21 Molecular Science and Biotechnology Institute, University of Melbourne, Parkville, VIC, Australia.; ^2^ Metabolomics Australia, Bio21 Molecular Science and Biotechnology Institute, University of Melbourne, Parkville, VIC, Australia.; ^3^ Murdoch Children’s Research Institute, Royal Children’s Hospital, Parkville, VIC, Australia.; ^4^ Department of Paediatrics, University of Melbourne, Parkville, VIC, Australia.; ^5^ Melbourne Mass Spectrometry and Proteomics Facility (MMSPF), Bio21 Molecular Science and Biotechnology Institute, University of Melbourne, Parkville, VIC, Australia.; ^6^ Victorian Clinical Genetics Services, Royal Children’s Hospital, Parkville, VIC, Australia.

## Abstract

During acute oxidative phosphorylation (OXPHOS) dysfunction, reversal of succinate dehydrogenase (complex II) maintains the redox state of the Coenzyme Q (Q)–pool by using fumarate as terminal electron acceptor in certain tissues and cell lines. We identified the action of SDHAF2 protein, a complex II assembly factor, as critical for metabolic adaptation during complex III dysfunction in HEK293T cells. SDHAF2 loss during complex III inhibition led to a net reductive TCA cycle from loss of succinate oxidation, loss of SDHA active site–derived reactive oxygen species (ROS) signaling, insufficient glycolytic adaptation, and a severe growth impairment. Glycolysis adapted cells, however, did not accumulate SDHAF2 upon Q-pool stress, exhibited a net reductive TCA cycle and mild growth phenotypes regardless of SDHAF2 presence. Thus, our study reveals how complex II assembly controls a balance between dynamics of TCA cycle directionality, protection from Q-pool stress, and an ability to use ROS-meditated signaling to overcome acute OXPHOS dysfunction in cells reliant on mitochondrial respiration.

## Teaser

Succinate dehydrogenase assembly dynamics maintains metabolic homeostasis during mitochondrial oxidative stress.

## INTRODUCTION

Mitochondrial respiratory chain complex II (CII), also known as succinate dehydrogenase (SDH), is an inner membrane-bound heterotetrametric (SDHA-D) enzyme that directly connects the tricarboxylic acid cycle (TCA) with oxidative phosphorylation (OXPHOS) and the production of adenosine 5′-triphosphate (ATP) (*1*–*3*). CII has been suggested to regulate cellular energy metabolism in scenarios such as cancer, diabetes, and inflammation due to its bidirectional interconversion of succinate to fumarate by the peripheral membrane catalytic subunit SDHA and ubiquinone (CoQ_10_) to ubiquinol (CoQ_10_H_2_) interconversion at a reaction site formed by membrane subunits SDHC and SDHD (*1*–*5*). SDHB connects these active sites and facilitates spontaneous bidirectional electron flux through three distinct iron-sulfur clusters, which are dynamically regulated based on the redox state of the ubiquinone to ubiquinol pool (Q-pool) (*6*–*8*). When CIII or CIV is inhibited, fumarate reduction via reversal of CII activity has been described as an alternative and adaptive mechanism to maintain canonical electron flow at CI and dihydroorotate dehydrogenase (DHODH) to support de novo pyrimidine biosynthesis and the detoxification of H_2_S because these events rely on an oxidized Q-pool (*6*, *7*). This phenomenon was shown to be tissue and cell type specific.

Human SDH assembly factor 2 (SDHAF2) and its homologs in yeast and bacteria are associated with the covalent flavination of SDHA (*9*–*12*). Knockout of the yeast (Sdh5) and bacterial (SdhE) homolog of SDHAF2 lead to an impairment of cellular respiration similar to loss of catalytic subunit SDHA (Sdh1/SdhA in yeast/bacteria) (*9*, *12*). While human SDHAF2 can complement Sdh5 in a Sdh5∆ yeast model, and heterozygous loss of function mutations in SDHAF2 are associated with hereditary paraganglioma (*12*), the complete loss of SDHAF2/SdhE in common human cancer-derived cell lines and thermophilic bacteria (*13*, *14*) does not lead to the expected loss of SDHA flavination. Furthermore, in human cell models, several stable long-lasting sub–100-kDa assemblies containing SDHA and either or both SDHAF2 and another CII assembly factor SDHAF4 accumulate under conditions where OXPHOS is compromised (*2*, *4*, *5*). Despite suggestions that these assemblies have a role in the homeostasis of pyrimidine synthesis during bioenergetic stress (*4*), recent in vitro and structural studies with purified proteins confirm that covalent flavination of SDHA is enhanced by the presence of SDHAF2 and requires a dicarboxylate such as fumarate, with flavination necessary for binding of SDHAF4 and later steps of CII assembly (*15*).

Here, using a panel of gene-edited human cell lines, each lacking a key subunit of OXPHOS CI to CV and quantitative proteomics, we found that SDHAF2 and the sub–100-kDa SDHA assemblies accumulate when assembly of OXPHOS CIII and CIV is defective. The accumulation of SDHAF2 can also be induced with small-molecule inhibitors of CII, CIII, and CIV and in cells cultured in hypoxic conditions. Spinelli *et al.* (*7*) recently demonstrated that cell lines and some mouse tissues exhibit net reversal of CII activity and use fumarate as terminal electron acceptor to protect the Q-pool redox state during hypoxia. Our results build on this by demonstrating that some cell lines avoid net reversal of the TCA cycle through accumulation of an SDHAF2-stabilized SDHA that protects the ability of mitochondria to signal a shift toward glycolytic metabolism through oxidation of succinate and CII-derived reactive oxygen species (ROS) by maintaining a canonical TCA cycle. The loss of SDHAF2 under normal conditions has a modest phenotype, consistent with it enhancing but not being essential for flavination of SDHA, while knockout under conditions of compromised OXPHOS leads to a reduction in ROS, consistent with the reduced capacity to flavinate SDHA and assemble mature CII, a severe impairment in cell proliferation, and a net reductive TCA cycle due to loss of canonical succinate oxidation. Thus, our study suggests that SDHAF2 enhancement of CII biogenesis controls a balance between protection of the Q-pool and succinate-derived ROS-mediated signaling necessary to cells reliant on mitochondrial OXPHOS.

## RESULTS

### Flavination of SDHA is required for maturation of CII and is enhanced by assembly factor SDHAF2

**Fig. 1.**
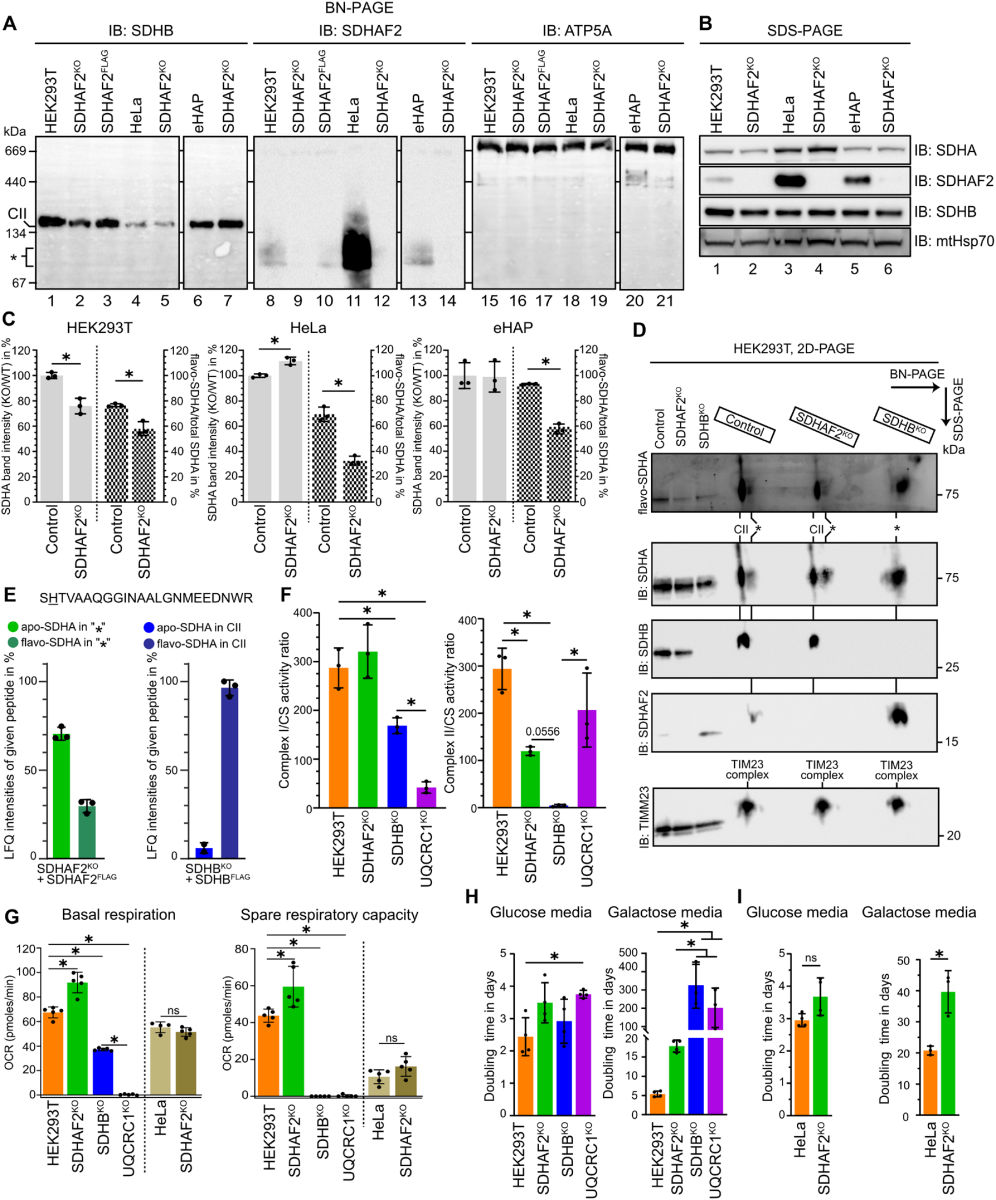
Flavination of SDHA is required for maturation of CII and is enhanced by SDHAF2. (**A**) BN-PAGE analysis of CII assembly in the indicated HEK293T, HeLa, or eHAP cell lines. Isolated mitochondria were solubilized in 1% (w/v) digitonin and complexes separated by BN-PAGE. SDHB and SDHAF2 containing complexes were detected by immunoblotting. * denotes the low–molecular weight stable subassemblies of CII. (**B**) Whole-cell lysates from the indicated HEK293T, HeLa, and eHAP cell lines were subjected to SDS-PAGE and immunoblotting using the indicated antibodies. (**C**) Quantification of relative band intensities of SDHA in knockout versus control (left axis) and comparison between flavo-SDHA and total SDHA (right axis). Means ± SD, *n* = 3 replicates. (**D**) Mitochondrial isolates from HEK293T, SDHAF2^KO^, and SDHB^KO^ cells analyzed by BN-PAGE, followed by SDS-PAGE in the second dimension. (**E**) Abundance of the indicated tryptic peptide containing FAD-modified His99 measured in elutes from FLAG^−^ immunoprecipitation of HEK239T SDHAF2^KO^ + SDHAF2^FLAG^ and SDHB^KO^ + SDHB^FLAG^ cells, expressed as % of total peptide. Means ± SD, *n* = 3 replicates. (**F**) Enzymatic activity of CI and CII was assessed in enriched mitochondrial fractions from the indicated HEK293T knockout cell lines. The activities (nmol/min/mg) are expressed as ratios to citrate synthase activity. Means ± SD, *n* = 3 replicates. (**G**) Basal oxygen consumption rate (OCR) and spare respiratory capacity of the indicated HEK293T and HeLa knockout cell lines. Means ± SD, *n* = 5 technical replicates per condition. (**H** and **I**) Growth comparisons of the indicated HEK293T and HeLa cell lines, measured as doubling time (in days), with either glucose- or galactose-containing media. Means ± SD; *n* = 4 replicates. Statistical significance (*P < 0.05) was determined using either a two-tailed Student’s *t* test [(C) and (I)] or a one-way analysis of variance (ANOVA) [(F) to (H)]. ns, not significant.

Despite original reports on the essentiality of SDHAF2 in flavination of CII catalytic subunit SDHA and its established role in heritable paraganglioma (*9*, *12*), CRISPR-Cas9–mediated knockout studies using popular human cancer-derived cell lines showed that flavination of SDHA occurs in its absence (*4*, *14*, *16*). We generated SDHAF2 knockouts in three different cell lines: the immortalized human embryonic kidney–derived HEK293T (human embryonic kidney–293T) cell line (*17*), eHAP (*18*), and HeLa (*19*) cell lines derived from chronic myelogenous leukemia and adenocarcinoma, respectively. The eHAP cells are notably an engineered haploid cell line derived from a subclone of the near-haploid KBM-7 cell line (*18*) and are frequently used in CRISPR-Cas9 screens. The loss of SDHAF2 expression was confirmed by blue native polyacrylamide gel electrophoresis (BN-PAGE), leading to a small but notable reduction in the amount of mature CII compared to control HEK293T cells (Fig. 1A, compare lanes 1 and 2) that is rescuable by reintroduction of SDHAF2^FLAG^ (Fig. 1A, lane 3) in line with other published SDHAF2^KO^ cell lines (*14*). Unexpectedly, no obvious reduction was noted in the abundance of mature CII in HeLa and eHAP SDHAF2^KO^ cells (Fig. 1A, compare lanes 4 and 5 and 6 and 7). We observed a relative increase in the basal levels of SDHAF2-containing subassemblies (denoted with “*” in Fig. 1A) in control HeLa cells and, to some degree, eHAP cells relative to HEK293T. We noted a similar trend in the steady-state levels of SDHAF2, with control HEK293T cells having the lowest abundance, followed by eHAP and HeLa cells (Fig. 1B, compare lanes 1, 3, and 5). Similar SDHAF2-containing low–molecular weight assemblies have been previously observed in MDA231 cells with impaired OXPHOS, leading to suggestions that these play a role in metabolic regulation during metastasis (*4*). We next assessed the flavination of SDHA in our SDHAF2^KO^ cell lines using an in-gel fluorescence assay that detects the autofluorescence of the covalently bound FAD (*12*, *14*, *20*). In agreement with previous studies (*13*, *14*), we observed flavinated-SDHA (flavo-SDHA) upon knockout of SDHAF2 across all three cell lines (fig. S1A and quantified in Fig. 1C). SDHAF2 loss led to a ~30 to 45% reduction in the amount of flavo-SDHA compared to unmodified SDHA (apo-SDHA) in all three backgrounds (Fig. 1C, checked bars). This only corresponded with a decrease in total SDHA in the HEK293T background, while SDHA levels in eHAP cells did not change and significantly increased in HeLa cells (Fig. 1C, gray bars). Similar trends in the abundance of assembled CII upon SDHAF2 loss were observed across cell lines (Fig. 1A).

To better understand the impact of SDHAF2 loss on CII, we repeated the in-gel fluorescence assay using two dimensional (2D)–PAGE (*21*) where the first dimension is BN-PAGE and the second SDS-PAGE. As shown in Fig. 1D, flavo-SDHA was found in both mature CII and sub–100-kDa assemblies in both control HEK293T cells and the SDHAF2^KO^ and accumulated in SDHB^KO^ cells where SDHA is unable to assemble into CII (*4*). We next sought to determine the ratio of flavo-SDHA to apo-SDHA in both mature CII and the sub–100-kDa assemblies. To do this, we used the SDHAF2^FLAG^ rescue of the HEK293T SDHAF2^KO^ described above (and additionally validated to rescue flavination of SDHA in fig. S1B) and an SDHB^FLAG^ rescue of the SDHB^KO^ (validated in fig. S1C). Using immunoprecipitation-coupled mass spectrometry analysis (IPMS), we show that SDHAF2^FLAG^ efficiently enriches SDHA but not other CII subunits (fig. S1D, left), while SDHB^FLAG^ enriches CII subunits but not assembly factors (fig. S1D, right). To quantify the amount of apo-SDHA and flavo-SDHA in IPMS eluates, we identified a tryptic peptide that contains the His^99^ residue modified with covalently attached flavin adenine dinucleotide (FAD) (fig. S1E). As can be seen in Fig. 1E (left), SDHAF2^FLAG^ eluates, representing SDHAF2-associated SDHA, contained a mixture of ~30% flavo-SDHA and ~70% apo-SDHA. In contrast, more than 90% of SDHA in SDHB^FLAG^ eluates, representing CII-associated SDHA, is flavinated (Fig. 1E, right). This result is in line with recent studies that demonstrate flavination of SDHA occurs before, and is likely necessary, for its assembly into mature CII (*2*).

Complex I and II play a crucial role in generating electrons required for OXPHOS. Thus, we next assessed CI and CII activity (Fig. 1F and fig. S1F) in HEK293T SDHAF2^KO^, the SDHAF2^FLAG^ rescue of SDHAF2^KO^, and SDHB and UQCRC1 knockout cell lines as controls for loss of CII and CI/III activity, respectively [noting that loss of UQCRC1 leads to CI deficiency due to defective CI assembly (*22*, *23*)]. As expected, we observed a significant loss of CI activity in the UQCRC1^KO^ line, but no such loss in cells lacking SDHAF2. A ~50% reduction in CI activity was seen in SDHB^KO^. Likewise, a complete loss of CII activity was observed in SDHB^KO^. The CII activity was reduced by two-thirds compared to control in SDHAF2^KO^ (Fig. 1F) in line with the observed reduced flavination of SDHA and mature CII (Fig. 1, A and C). CII activity was rescued in SDHAF2^FLAG^ cells (fig. S1F) and UQCRC1^KO^ cells exhibited a one-third reduction in CII activity compared to control (Fig. 1F). To assess the effect of SDHAF2 loss on mitochondrial respiration, we next measured oxygen consumption rates (OCRs). Unexpectedly, in the HEK293T background, SDHAF2^KO^ cells exhibited a nearly one-third increase in basal respiration relative to control (Fig. 1G and fig. S1G). In contrast, SDHB^KO^ cells experienced a significant reduction in basal respiration compared to control, while UQCRC1^KO^ showed no detectable levels of respiration, as we have shown previously (*23*). Similar trends were seen in spare respiratory capacity with the exception of SDHB^KO^, which had no spare capacity (Fig. 1G and fig. S1G). In contrast, there were no significant differences in basal and spare respiratory capacity between control and HeLa cells lacking SDHAF2; however, spare respiratory capacity is noticeably lower in the HeLa background compared to HEK293T, suggesting that mitochondrial respiration is already at a maximum in HeLa cells, while HEK293T have an ability to dynamically increase respiration.

To investigate the effects on cell proliferation arising from OXPHOS dysfunction due to the loss of SDHAF2, we next evaluated the growth rate of cells using live-cell bright-field imaging. Cells were cultured in either normal glucose-containing media or media containing galactose in place of glucose, which is known to require a functional OXPHOS system to maintain cell viability (*24*). The proliferation rates of cell lines were monitored for up to a week (fig. S1H) to calculate growth rate (Fig. 1, H and I). As expected, control HEK293T cells exhibited shorter doubling times in glucose compared to galactose media (Fig. 1H). The knockout of UQCRC1 in the HEK293T background demonstrated a minor growth impairment in glucose media but did not grow in galactose media (fig. S1H). Neither SDHAF2^KO^ of SDHB^KO^ had significant growth defects in glucose media, and when cultured in galactose, SDHAF2^KO^ displayed only a modest reduction in growth rate compared to control cells whereas SDHB^KO^ behaved similarly to UQCRC1^KO^. Loss of SDHAF2 in HeLa cells resulted in a similarly moderate growth phenotype as was observed in the HEK293T background in both glucose and galactose media (Fig. 1I). The loss of CIII activity in HeLa cells (treated with antimycin A as a proxy for knockout of UQCRC1 in the HEK293T model) similarly had a moderate phenotype in glucose media, while cell death was observed 8 hours after cells were transferred to galactose media in the presence of antimycin A (fig. S1I). Together, we conclude that the absence of SDHAF2 results in a mild growth defect which we attribute to a reduction but not complete loss of CII activity, consistent with reports suggesting that this protein is not required but enhances flavination of SDHA (*13*, *14*). Our data also support recent conclusions from in vitro models suggesting that flavination of SDHA occurs during SDHAF2 binding and is likely essential for its incorporation into mature CII (*2*).

### The SDHAF2-SDHA subassembly accumulates during defective OXPHOS in HEK293T cells

**Fig. 2.**
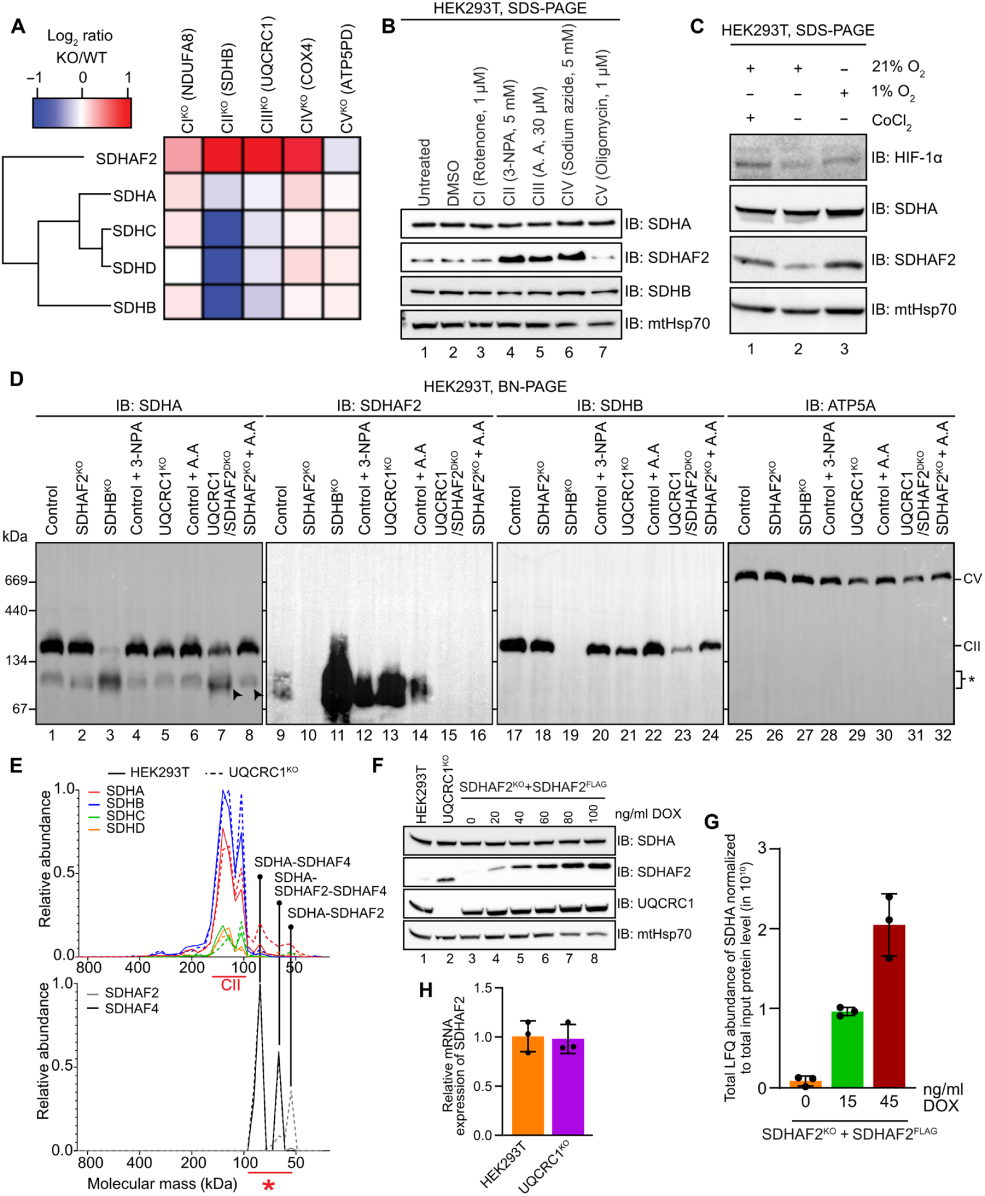
The SDHAF2-SDHA subassembly accumulates during defective OXPHOS in HEK293T cells. (**A**) Heatmap representing SILAC MS-derived ratios of CII subunits and SDHAF2 in the indicated HEK293T knockout cell lines compared to HEK293T cells. (**B**) Inhibition of CII and CIII lead to elevation of SDHAF2 in HEK293T cells. Cells were individually treated with indicated inhibitors or DMSO for 8 hours. Whole-cell lysates were subjected to SDS-PAGE and immunoblotting using the indicated antibodies. (**C**) HEK293T cells were cultured under normoxic (21% O_2_) or hypoxic conditions (1% O_2_) or media supplemented with 100 μM CoCl_2_ for 24 hours. Whole-cell lysates were analyzed by SDS-PAGE and immunoblotting using the indicated antibodies. (**D**) BN-PAGE analysis of CII and its low molecular weight sub-assemblies (denoted by *) in mitochondria isolated from the indicated cell lines treated as indicated. BN-PAGE gels were subjected to immunoblotting using the indicated antibodies. (**E**) Complexome profiling of CII and its subassemblies in control HEK293T (solid lines) and UQCRC1^KO^ (dotted lines) mitochondria isolated from SILAC-labeled cells. The plots show relative intensities of heavy- and light-labeled proteins averaged across all CII subunits (top) or assembly factors (bottom), with the maximum set as 1. Molecular mass of each complex was calculated as described in Materials and Methods. (**F**) SDS-PAGE analysis of cell lysates from HEK293T SDHAF2^KO^ cells expressing SDHAF2^FLAG^ under the control of a doxycycline-inducible Tet promoter. (**G**) Assessment of total abundance of SDHA co-immunoprecipitated during IPMS from HEK293T SDHAF2^KO^ + SDHAF2^FLAG^ cells expressing different levels of SDHAF2. Means ± SD, *n* = 3 biological replicates. (**H**) Transcript levels in HEK293T and UQCRC1^KO^ cells. Means ± SD, *n* = 3 technical replicates.

We and others have identified assembly factors for the respiratory chain complexes based on their up-regulation following knockout or mutation of subunits and related proteins (*24*, *25*). We assembled a panel of knockout HEK293T cell lines, each lacking a key subunit of the five OXPHOS complexes (NDUFA8 for CI, SDHB for CII, UQCRC1 for CIII, COX4 for CIV, and ATP5PD for CV), resulting in their defective assembly (fig. S2A). To identify changes in the cellular proteome of each knockout, we used stable isotope labeling using amino acids in cell culture (SILAC) followed by quantitative proteomics (fig. S2B and data S1). Interestingly, and somewhat unexpectedly, in CII, CIII, and CIV knockouts, we found a significant increase in steady-state levels of SDHAF2 (Fig. 2A and figs. S2, A and B), which correlated with an increased accumulation of SDHAF2-containing sub–100-kDa assemblies (fig. S2B). In each of these knockouts, SDHAF2 was one of the proteins most increased in abundance relative to controls across the cellular proteome (fig. S2B and data S1). Notably, no significant changes in the abundance of CII subunits, including SDHA, were observed in any cell line, except for in SDHB^KO^ due to impaired CII assembly. We also did not observe significant changes in the abundance of any other CII assembly factor, with the exception of an increase in SDHAF4 levels in the CIII knockout (UQCRC1^KO^; fig. S2B).

We next investigated if accumulation of SDHAF2 occurs following nongenetic impacts to the OXPHOS system. First, we performed immunoblotting analysis of control HEK293T cells treated with different well-characterized OXPHOS inhibitors for 8 hours (*26*–*28*). Unexpectedly, the abundance of SDHAF2 was increased following inhibition of CII with 3-nitropropionic acid (3-NPA), CIII with antimycin A, and CIV with sodium azide but not the inhibition of CI or CV (rotenone and oligomycin, respectively) (Fig. 2B). In the case of antimycin A treatment, we found increased levels of SDHAF2 to be dose dependent (fig. S2C, compare lanes 2 to 4 with lane 1). We next asked if growth under hypoxia, which is known to suppress OXPHOS (*29*, *30*), similarly resulted in increased levels of SDHAF2. To achieve this, we exposed cells to 1% oxygen for 24 hours followed by immunoblotting. Low oxygen resulted in the stabilization of hypoxia-inducible factor–1α (HIF-1α) (Fig. 2C), as expected, and led to an increased abundance of SDHAF2. Incubation of cells with cobalt (II) chloride, which is known to stabilize HIF-1α and induce a hypoxia-like response (*31*), also resulted in an increase in SDHAF2 levels.

Using BN-PAGE, we noted that both knockout and inhibition of CIII resulted in accumulation of the sub–100-kDa assemblies previously described to contain SDHA and one or both of SDHAF2 and SDHAF4 (*4*) (denoted as * in Fig. 2D). As with previous experiments, BN-PAGE analysis showed that loss of SDHAF2 led to small reductions in the amount of mature CII and revealed altered distribution of sub–100-kDa SDHA-containing complexes, suggesting accumulation of free SDHA (labeled with an arrowhead in Fig. 2D) as previously described in hPheo1 SDHAF2^KO^ cells (*2*). To better resolve and quantify the abundance of each complex in cells accumulating sub–100-kDa SDHA-containing assembles, we turned to complexome profiling, which combines high-resolution BN-PAGE with quantitative MS (*21*). Complexome analysis resolved the distribution of CII subunits and assembly factors into discrete complexes. As can be seen in Fig. 2E and data S2, we identified all three previously identified (*2*) sub–100-kDa assemblies in control HEK293T and UQCRC1^KO^ cells: SDHA-SDHAF4 (~82.4 kDa), SDHA-SDHAF2-SDHAF4 (~64.4 kDa), and SDHA-SDHAF2 (~54.6 kDa). Notably, when comparing the profile plots of UQCRC1^KO^ and control cells, we observed an increase in abundance of the ~55 kDa SDHA-SDHAF2 subassembly, while no free SDHA was observed. We hypothesized that free SDHA is only stable in association with SDHAF2 and/or SDHAF4 or in the mature CII. To test this, we used the SDHAF2^FLAG^ rescue cell line, where SDHAF2^FLAG^ expression is doxycycline (DOX) inducible (Fig. 2F) coupled with IPMS analysis and increasing concentrations of DOX. We observed increasing enrichment of SDHA in line with increased expression of SDHAF2^FLAG^ (Fig. 2G). This result supports a codependence between SDHAF2 and SDHA and is consistent with previous observations that free SDHAF2 is protected from continual proteolysis by mitochondrial protease LONP1 through its stable interaction with SDHA (*32*). In support of this, real-time quantitative polymerase chain reaction on control and UQCRC1^KO^ cell lines showed no change in SDHAF2 transcript levels (Fig. 2H) suggesting that the increased abundance of SDHAF2 protein in the CIII knockout is due to reduced proteolysis. Together, we conclude that SDHAF2 protein and a sub–100-kDa assembly containing SDHAF2 and SDHA accumulate during dysfunction of Complexes II, III and IV, and except for in the absence of SDHB, this is not a consequence of aberrant protein complex assembly. Further, as sequestration of SDHA by SDHAF2 under these conditions does not appear to impact total SDHA levels in HEK293T cells, this suggests that both SDHA and SDHAF2 are unstable when not in complex with each other or other CII-associated proteins and that increased levels of the SDHA-SDHAF2 assembly may represent increased CII biogenesis.

### Loss of SDHAF2 during CIII dysfunction leads to a severe growth defect and net reversal of the TCA cycle in HEK293T cells

**Fig. 3.**
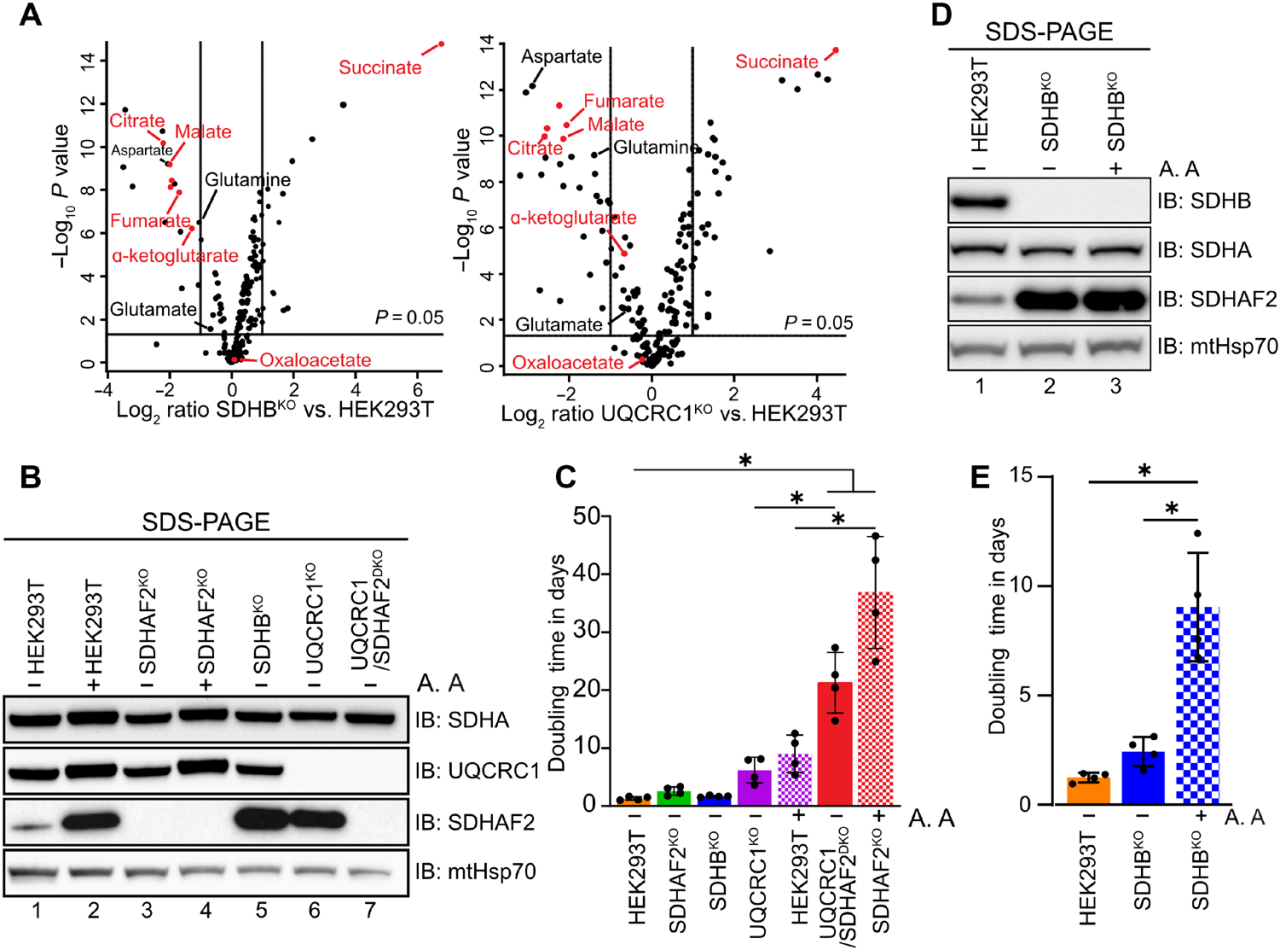
Loss of SDHAF2 compounded with CIII dysfunction leads to a severe growth defect in HEK293T cells. (**A**) Steady-state levels of polar metabolites in HEK293T, SDHB^KO^, and UQCRC1^KO^ compared to WT cells, *n* = 6 biological replicates. Red colored dots indicate TCA intermediates. The horizontal line indicates *P* = 0.05 and the vertical line a fold change of 2. *P* values were calculated using a two-tailed single sample *t* test. (**B**) At the conclusion of the growth assay in (C), representative cell lysates were analyzed by SDS-PAGE and immunoblotting using indicated antibodies. (**C**) Growth comparison of the indicated HEK293T cell lines, measured as doubling time (in days), with or without antimycin A. Means ± SD, *n* = 4 technical replicates. *P < 0.05. *P* values were calculated using a one-way ANOVA. (**D**) As for (B) but using representative cell lines from (E). (**E**) Growth comparison of HEK293T and SDHB^KO^ cells treated with antimycin A, as described in (A). Means ± SD, *n* = 4 technical replicates. *P < 0.05. *P* values were calculated using a one-way ANOVA.

It is well known that succinate accumulates following acute OXPHOS insults such as hypoxia (*33*, *34*), ischemic heart attack (*35*), and in muscle postexercise (*36*). In line with this, steady-state gas chromatography MS (GC-MS) of polar metabolites in the HEK293T UQCRC1^KO^ cell line revealed a significant increase in succinate and a similar behavior in respect to the abundances of other TCA metabolites as was observed in SDHB^KO^ (Fig. 3A and data S3). To investigate this further, we generated a double knockout of SDHAF2 and UQCRC1 (UQCRC1/SDHAF2^DKO^) in the HEK293T UQCRC1^KO^ background (Fig. 2D, lanes 7, 15, and 23). We also inhibited CIII with antimycin A in SDHAF2^KO^ cells to assess the immediate consequences of OXPHOS stress on SDHAF2 levels (Fig. 3B) and cell proliferation in glucose-containing media (Fig. 3C and fig. S3A). As expected, we observed similar moderate growth defects in control HEK293T cells treated with antimycin A and untreated UQCRC1^KO^ cells. Intriguingly, SDHAF2^KO^ cells grown in media containing antimycin A and our untreated UQCRC1/SDHAF2^DKO^ cells displayed similar severe growth impairment, characterized by the highest doubling time among all assessed cell lines (Fig. 3C and fig. S3A). Next, we asked if the compounded growth defect of SDHAF2 loss in the presence of CIII dysfunction could be due to the moderate impact SDHAF2 loss has on CII activity (Fig. 1F) and proliferation (Figs. 1H and 3C). To test this, we repeated the growth assay in SDHB^KO^ cells (which have no detectable CII activity; Fig. 1F) in the presence and absence of antimycin A. Immunoblot analysis of SDHB^KO^ cells revealed an elevated level of SDHAF2 (Fig. 3D) as seen previously. Subsequent inhibition of CIII through addition of antimycin A did not lead to an increase in the steady-state level of SDHAF2 above what is found in untreated SDHB^KO^ cells. While treatment of SDHB^KO^ with antimycin A did result in a significant growth reduction compared to loss of CII activity alone (Fig. 3E and fig. S3B), this was far less severe than seen in SDHAF2^KO^ cells treated with antimycin A or the UQCRC1/SDHAF2^DKO^ cell line (doubling time of ~9 days versus ~20 to 35 days; see Fig. 3, C and E).

**Fig. 4.**
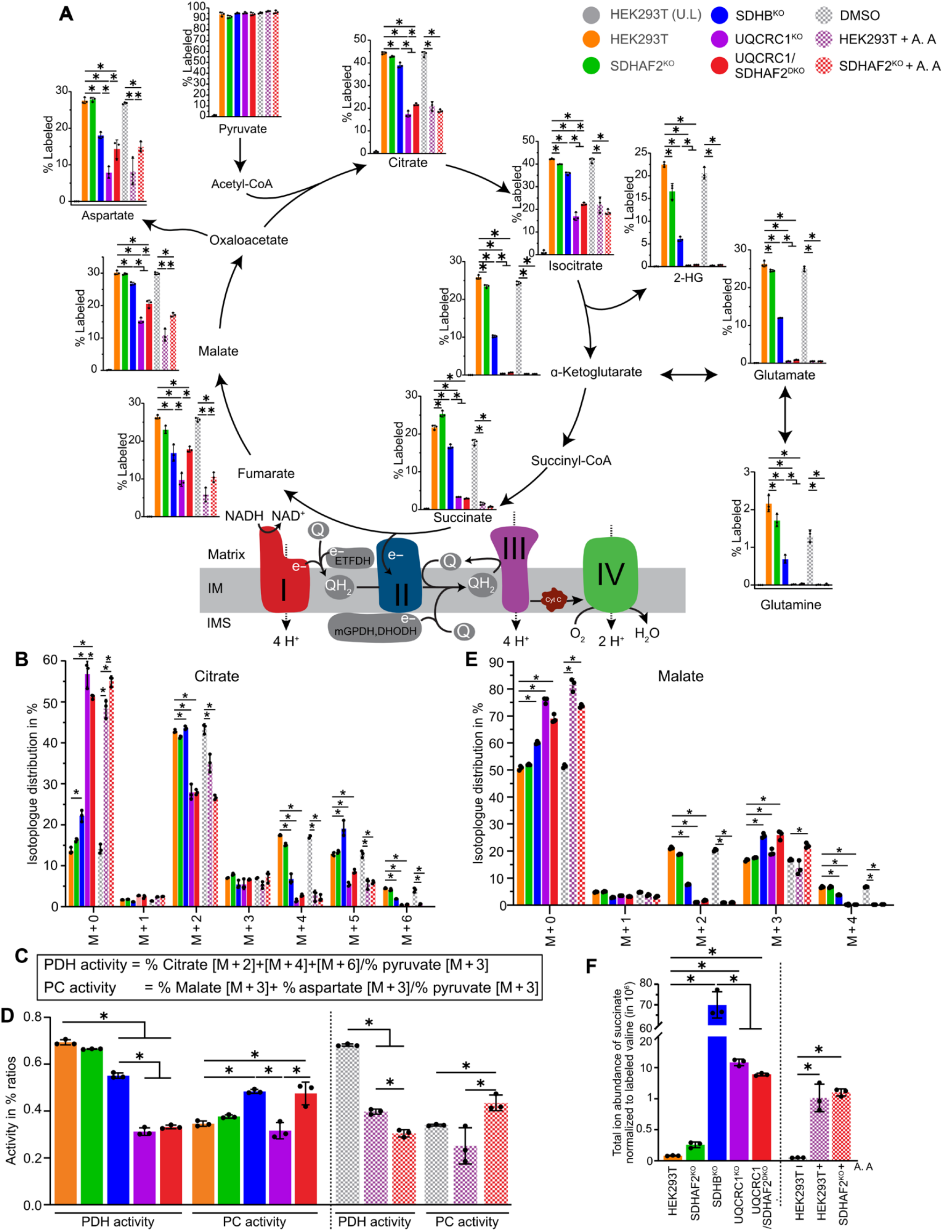
Inhibition of CIII compounded with loss of SDHAF2 leads to altered carbon utilization in HEK293T cells. (**A**) Percentage labeling of TCA metabolites with ^13^C in WT HEK293T, SDHAF2^KO^, SDHB^KO^, UQCRC1^KO^, and UQCRC1/SDHAF2^DKO^ cells following a 24-hour incubation in growth media either supplemented with 25 mM ^13^C_6_-glucose or unlabeled (U.L.). Hatched bars indicate treatment with DMSO or antimycin A starting 8 hours before labeling experiment. (**B**) Mass isotopolog distribution of citrate after natural abundance correction. (**C**) Equations used in calculation of PDH and PC activities using percentage of differently labeled citrate, malate, and aspartate produced from fully labeled pyruvate during ^13^C_6_-glucose tracing. (**D**) PDH and PC activities in HEK293T cell lines from stable isotope tracing of 25 mM ^13^C_5_-glucose using the equation outlined in (C). Cell lines and conditions are indicated in (A). (**E**) As for (B) but showing the mass isotopolog distribution for malate. (**F**) Abundance of total succinate after a 24-hour tracer experiment. All data presented as means ± SD following natural abundance corrections, *n* = 3 biological replicates. *P < 0.05. *P* values were calculated using a one-way ANOVA.

To investigate this further, we assessed carbon flux through the TCA cycle using in vitro stable isotope tracer metabolomics with ^13^C_6_-glucose. OXPHOS-defective HEK293T cells with either elevated levels of SDHAF2 (SDHB^KO^, UQCRC1^KO^, and control cells treated with antimycin A) or lacking SDHAF2 entirely (SDHAF2^KO^, UQCR-C1/SDHAF2^DKO^, and SDHAF2^KO^ treated with antimycin A) were incubated in media supplemented with 25 mM ^13^C_6_-glucose for 24 hours. Polar metabolites were extracted and analyzed using liquid chromatography–MS (LC-MS). ^13^C-enrichment in TCA cycle intermediates was significantly reduced in cells with either a genetic (UQCRC1^KO^ and UQCRC1/SDHAF2^DKO^) or antimycin A–based OXPHOS dysfunction regardless of the presence of SDHAF2 (Fig. 4A, compare purple and red shaded and hatched bars to the orange bar). Further detailed analysis of the isotopomer distribution in key TCA cycle intermediates revealed a decrease in isotopomers associated with operation of a canonical cyclic TCA in which carbon backbones cycle multiple times (e.g., leading to M + 2, M + 4, and M + 6 citrate isotopomers and M + 2 and M + 4 malate isotopomers) with a concomitant increase in the unlabeled (M + 0) isotopomer of all TCA intermediates (Fig. 4, B and E and others in fig. S4). Although SDHB^KO^ cells followed a similar trend, the impact was relatively modest and consistent with their modest basal respiration rates compared to UQCRC1^KO^ (see Fig. 1D). SDHAF2^KO^ cells had labeling profiles similar to control cells, further supporting our data and the literature (*4*, *14*) that suggests that SDHAF2 is not essential and that its loss is tolerated in HEK293T cells under normal growth conditions in glucose media.

The TCA cycle can proceed in two distinct directions. The first is the canonical and thermodynamically favored oxidative cycle, which leads to the generation of reducing equivalents that drive the respiratory chain and OXPHOS (*37*, *38*). Alternatively, under certain nutrient or stress conditions, such as when OXPHOS is defective, key reactions in the TCA cycle can proceed in the reverse direction to form a reductive cycle (*33*, *39*, *40*). Pyruvate dehydrogenase (PDH) and pyruvate carboxylase (PC) are distinct enzymes that facilitate the entry of glucose-derived pyruvate into the TCA cycle under different conditions. PDH catalyzes the conversion of pyruvate into acetyl–coenzyme A (CoA), which feeds carbon into the oxidative (forward) arm of the TCA cycle, whereas PC catalyzes the carboxylation of pyruvate into oxaloacetic acid (OAA) during anaplerosis or following a switch to the reductive or reverse TCA cycle. During each round of oxidative (forward) TCA cycle, M + 2 acetyl-CoA derived from ^13^C_6_-glucose combines with OAA to form M + 2 citrate with more highly labeled isotopomers (e.g., M + 4 and M + 6) forming with subsequent cycles. In contrast, during reductive (reverse) TCA cycle, PC catalyzes the carboxylation of fully labeled M + 3 pyruvate to form M + 3 OAA, which is subsequently converted into either M + 3 malate or M + 3 aspartate (through transamination of OAA). Hence, the relative ratio of M + 2/M + 4/M + 6 citrate to M + 3 pyruvate and the ratio of combined M + 3 malate/M + 3 aspartate to M + 3 pyruvate can be used to assess PDH and PC activity, respectively (Fig. 4C). As seen in Fig. 4D, we observed a correlation between the activities of PDH and PC in cells with and without CIII dysfunction. Specifically, UQCRC1^KO^ and antimycin A–treated control cells, along with the UQCRC1/SDHAF2^DKO^ and antimycin A–treated SDHAF2^KO^ cells, displayed reduced PDH activity relative to untreated control and SDHAF2^KO^ cells, consistent with a reduced oxidative TCA cycle flux. However, in the absence of SDHAF2 (i.e., UQCRC1/SDHAF2^DKO^ and antimycin A–treated SDHAF2^KO^ cells), we observed a significant increase in PC activity over what is observed in the presence of SDHAF2 (i.e., UQCRC1^KO^ and antimycin A–treated cells), suggesting that these cells have increased carbon flux in the reverse direction. The loss of SDHB and complete absence of SDH activity also results in increased PC activity (Fig. 4D), although the reduction of PDH activity was not as large as seen in UQCRC1/SDHAF2^DKO^ and antimycin A–treated SDHAF2^KO^ cells. While SDHB^KO^ cells initially displayed a level of M + 2 incorporation into citrate that closely resembled that of control cells, they failed to generate a comparable amount of M + 4 and M + 6 labeled citrate indicating limited cycling around an oxidative TCA cycle (Fig. 4B, blue bar). As SDHB^KO^ cells also had a significant reduction of M + 2 malate (Fig. 4E), the low levels of M + 4 and M + 6 citrate and M + 2 malate may reflect the nonenzymatic oxidation of succinate into fumarate due to its accumulation in these cells (Fig. 4F).

Increased reductive (reverse) TCA flux in cells with impaired OXPHOS and lacking SDHAF2 was further supported by increased abundance of M + 3 fumarate (derived from M + 3 malate) relative to the comparable cell line expressing SDHAF2 (fig. S4). However, M + 3 isotopomers of other TCA cycle intermediates, such as succinate, were not detected. The substantial increase in the total intracellular pool of succinate in OXPHOS functional cells (in the background of UQCRC1^KO^ cells or through antimycin A treatment) over control cells is comparable regardless of the presence or absence of SDHAF2 (Fig. 4F, compare purple and red solid and checked bars). Further, given >90% of succinate under these conditions is M + 0 (fig. S4, top right, compare M + 0 purple and red solid and checked bars), this suggests that another carbon source is being used. This may therefore reflect increased glutaminolysis and flux through the oxidative arm of the TCA cycle from α-ketoglutarate to succinate (*33*, *34*, *39*).

**Fig. 5.**
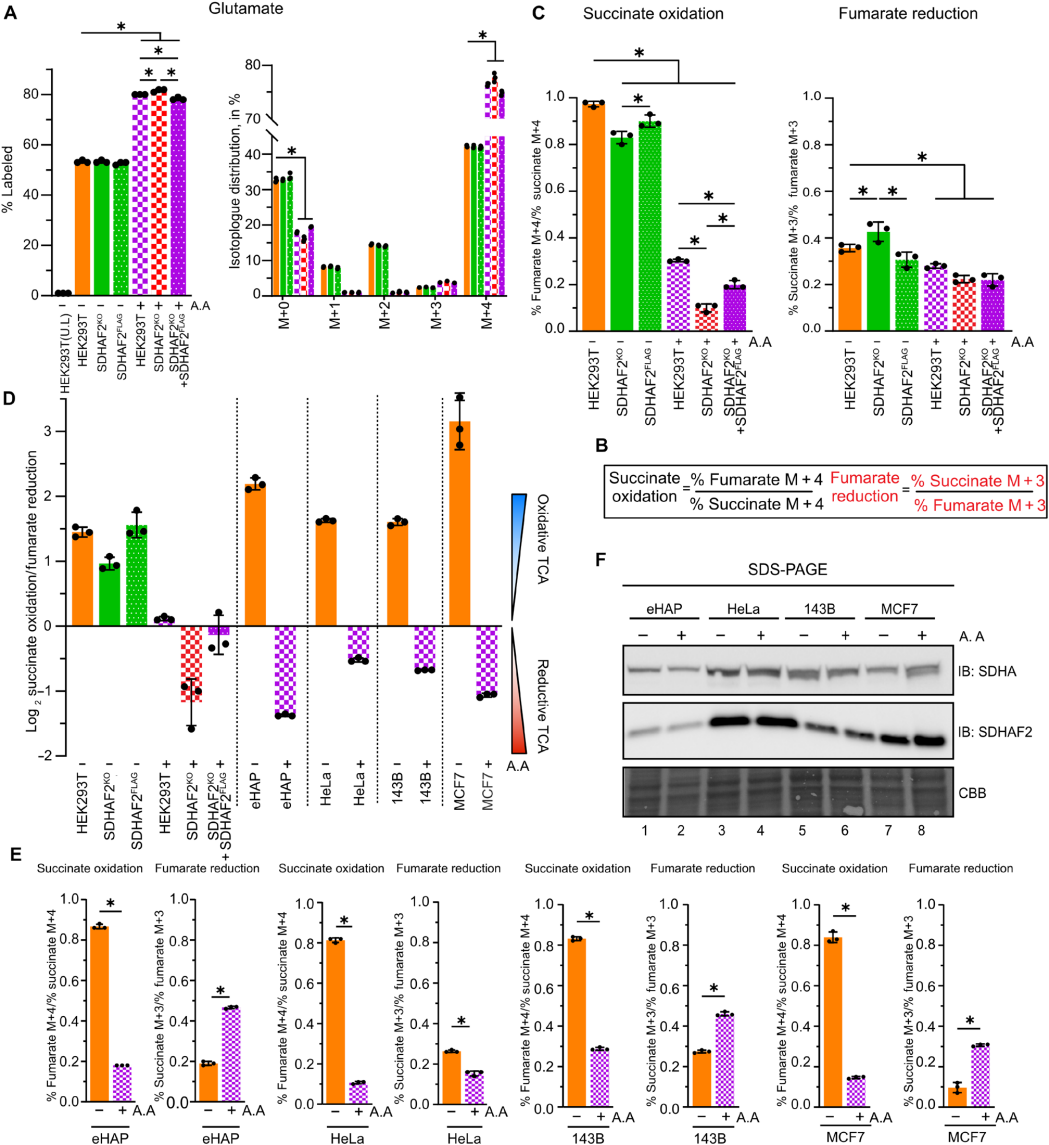
Effect of CIII inhibition on TCA cycle directionality. (**A**) Mass isotopolog distribution of glutamate after natural abundance correction, along with percentage labeling, in control HEK293T, SDHAF2^KO^, and SDHAF2^KO^ + SDHAF2^FLAG^ cells, with and without 8-hour antimycin A treatment, following an 8-hour incubation in growth media supplemented with either 2 mM ^13^C_5_-glutamine or unlabeled glutamine. (**B**) Equations used in calculation of succinate oxidation via forward activity of CII and fumarate reduction via reverse activity of SDH using the percentage of differently labeled succinate and fumarate produced during ^13^C_5_-glutamine tracing. Succinate oxidation activity is measured using the ratio of percent labeled fumarate M + 4 to percent labeled succinate M + 4. Fumarate reduction activity is measured using the ratio of percent labeled succinate M + 3 to percent labeled fumarate M + 3. (**C**) Succinate oxidation and fumarate reduction as determined from stable isotope tracing of 2 mM ^13^C_5_-glutamine using the equation outlined in (B). (**D**) Net TCA cycle direction based on the log_2_-transformed ratio of succinate oxidation to fumarate reduction in cell lines subjected to ^13^C_5_-glutamine tracing analysis. (**E**) Measurement of succinate oxidation and fumarate reduction as given in (C). (**F**) SDHAF2 response to antimycin A in eHAP, HeLa, 143B, and MCF7 cells. Cells were treated with antimycin A or DMSO for 8 hours. Cell lysates were subjected to SDS-PAGE and immunoblotting using indicated antibodies. All data presented as means ± SD following natural abundance corrections, *n* = 3 biological replicates. *P < 0.05. *P* values were calculated using either a one-way ANOVA [(A) and (C)] or a two-tailed Student’s *t* test (E). CBB, Coomassie Brilliant Blue R.

To investigate this further, increased glutaminolysis and flux into both the oxidative and reductive arms of the TCA cycle in cells with defective CIII was assessed by labeling cells with 2 mM ^13^C_5_-glutamine for 8 hours followed by targeted GC-MS (Fig. 5A). For these experiments, we used antimycin A to induce OXPHOS dysfunction and exclude the pleotropic effects of defective CIII assembly in UQCRC1^KO^ that includes misassembly of CI and CIV (fig. S2A). Glutamine labeling revealed significantly increased levels of labeled glutamate, with up to 80% of the total population being M + 4 labeled in cells treated with antimycin A, including SDHAF2^KO^ cells (Fig. 5A).

Quantitation of the ratio of M + 4 and M + 3 isotopomers of succinate and fumarate can be used to calculate the net direction of the TCA cycle at CII (*7*). Succinate oxidation (forward direction) in ^13^C_5_-glutamine–fed cells can be quantified from the ratio of M + 4 fumarate/M + 4 succinate, while fumarate reduction (reverse direction) can be quantified from the ratio M + 3 succinate/M + 3 fumarate (Fig. 5B). Because of technical limitations resolving succinate and fumarate peaks, we switched from targeted to a multiple reaction monitoring (MRM) approach. We noted a decrease in succinate oxidation of ~60% in HEK293T cells treated with antimycin A compared to untreated cells, consistent with a significant reduction in forward SDH activity. Subsequent loss of SDHAF2 (i.e., SDHAF2^KO^ treated with antimycin A) resulted in a further decrease in succinate oxidation (Fig. 5C, compare purple and red checked bars) and therefore strong inhibition of forward SDH activity (an additional ~30%, consistent with the loss of SDH activity from impaired SDHA flavination; see Fig. 1, C and F) that is rescued by reintroduction of SDHAF2^FLAG^. In contrast, in the presence of antimycin A, fumarate reduction did not change upon SDHAF2 loss, suggesting that fumarate reduction under these conditions is nonenzymatic, consistent with previous literature (*7*).

Spinelli *et al.* (*7*) recently reported that inhibition of CIII with antimycin A or knockout of UQCRC2 or knockout of CIV subunit COX4 results in a net reductive TCA cycle in osteosarcoma-derived 143B cells (*7*). We assessed net TCA directionality in 143B cells, our eHAP and HeLa models, and the adenocarcinoma breast cancer cell line MCF7. All four cell lines exhibited net reductive TCA upon antimycin A treatment (Fig. 5, D and E). Unexpectedly, SDHAF2 levels remained unchanged in all four tumor-derived cell lines following antimycin A treatment (Fig. 5F). The preference for glycolysis in these cell lines is well known. In our hands, HeLa cells grow poorly in galactose compared to HEK293T cells (fig. S1H) and have lower basal OCR and spare respiratory capacity (Fig. 1G). Further, the ratio of SDHAF2-containing sub–100-kDa assemblies (Fig. 1A) to mature CII is much higher in HeLa cells compared to HEK293T cells. Given that the accumulation of SDHAF2 in HEK293T cells is not a transcriptional response (Fig. 2H), this suggests that HeLa and likely the other tumor-derived cell lines may be unable to dynamically increase CII biogenesis. Together, in HEK293T cells, following treatment with antimycin A, the loss of SDHAF2 leads to a net reductive TCA cycle (Fig. 5D), which we attribute to a reduced capacity to increase CII biogenesis.

### Altered ubiquinol/ubiquinone ratio drives SDHAF2-SDHA accumulation in HEK293T cells during CIII dysfunction

**Fig. 6.**
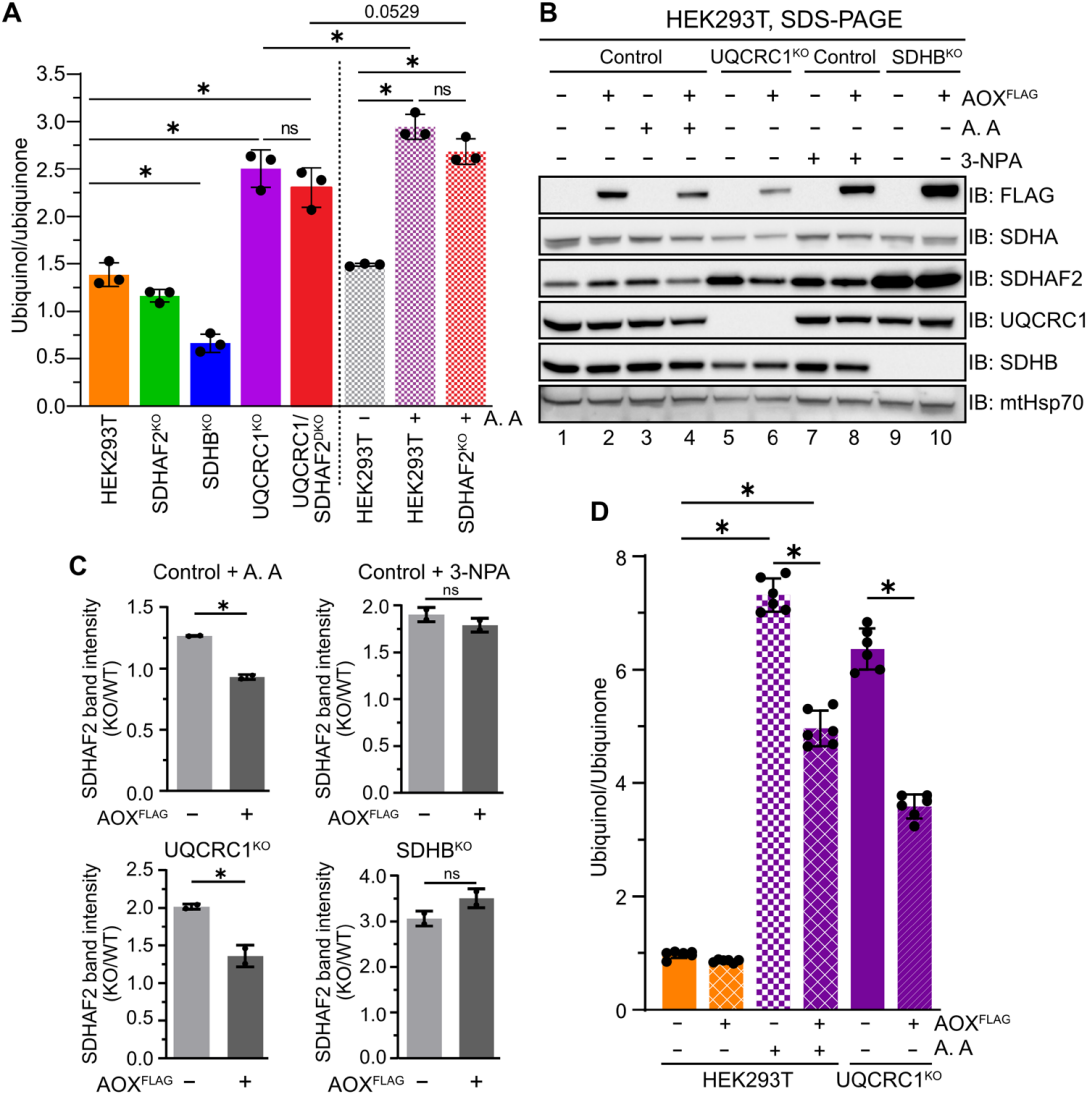
Altered ubiquinol/ubiquinone ratio drives SDHAF2-SDHA accumulation in HEK293T cells during CIII dysfunction. (**A**) Ratiometric abundances of ubiquinol to ubiquinone as measured by targeted LC-MS/MS on whole-cell lipid extracts from the indicated HEK293T cell lines. Hatched bars indicate treatment with DMSO or antimycin A 8 hours before lipid extraction. (**B**) Expression of AOX targeted to the inner mitochondrial membrane can reverse accumulation of antimycin A–induced accumulation of SDHAF2. Expression of AOX was induced for 24 hours as indicated in the indicated HEK293T cell lines followed by 8 hours of treatment with or without 3-NPA or antimycin A. Cell lysates were subjected to SDS-PAGE and immunoblotting using the indicated antibodies. (**C**) SDHAF2 band intensities in knockout HEK293T cells from (B), and replicate experiments were quantified and normalized to the SDHAF2 band in the comparable WT cell line. Means ± SD, *n* = 2 replicates. *P < 0.05. *P* values were calculated using a two-tailed Student’s *t* test. (**D**) Measurement of ubiquinol to ubiquinone ratio in cells with or without expression of AOX as indicated, as described in (A). *P < 0.05. *P* values were calculated using a one-way ANOVA.

In addition to TCA directionality, Spinelli *et al.* (*7*) also observed an altered ubiquinol/ubiquinone ratio in antimycin A–treated 143B cells (*7*). We quantified the abundance of ubiquinol and ubiquinone using LC-MS/MS (*41*) in both UQCRC1^KO^ and antimycin A–based models of OXPHOS dysfunction in the HEK293T background. As can be seen in Fig. 6A, cells exhibiting prominent succinate oxidation indicative of forward SDH activity (in control and SDHAF2^KO^) displayed a 1.4-fold ratio of ubiquinol (CoQ_10_H_2_) to ubiquinone (CoQ_10_). As expected, UQCRC1^KO^ and antimycin A–treated cells exhibited an increased ubiquinol to ubiquinone ratio, consistent with a reduced capacity of CIII to oxidize ubiquinol. SDHB^KO^ has no SDH activity (Fig. 1F), and as expected, this results in a lower ubiquinol to ubiquinone ratio (Fig. 6A), which suggests that in HEK293T cells CII is a major contributor to the Q-pool. HEK293T cells with net reductive TCA due to loss of SDHAF2 and antimycin A treatment, as well as UQCRC1^KO^ and UQCRC1/SDHAF2^DKO^, showed a slight trend toward a lower ubiquinol to ubiquinone ratio relative to conditions where SDHAF2 is present; however, the change in ratio was not significant (compare red solid/hatched to purple solid/hatched in Fig. 6A). These results suggest that changes in the ratio of ubiquinol/ubiquinone do not underlie the severe growth defect observed in OXPHOS deficient HEK293T cells lacking SDHAF2 (Fig. 3C and fig. S3A).

To better understand the connection between SDHAF2 levels and maintenance of the Q-pool, we expressed a FLAG tagged alternative oxidase (AOX) in control HEK293T, SDHB^KO^, and UQCRC1^KO^ cells. This fusion protein is targeted to the inner mitochondrial membrane and is capable of oxidizing ubiquinol to ubiquinone in an antimycin A–independent manner (*7*, *8*) (Fig. 6B). Consistent with our hypothesis, the expression of AOX resulted in a significant reduction of SDHAF2 protein abundance following the inhibition of CIII in either antimycin A–treated control HEK293T or UQCRC1^KO^ cells (Fig. 6B comparing lane 4 to 3 and lane 6 to 5, quantified in Fig. 6C). Furthermore, the high ubiquinol/ubiquinone ratio in antimycin A–treated control cells or UQCRC1^KO^ cells was lowered by the expression of AOX (Fig. 6D). Unexpectedly, the expression of AOX in 3-NPA–treated control and SDHB^KO^ cells did not reduce the levels of SDHAF2 (Fig. 6B, compare lane 8 to 7 and lane 10 to 9, quantified in Fig. 6C). Because loss of CII activity in SDHB^KO^ also did not increase the ubiquinol to ubiquinone ratio (Fig. 6A), and in this cell line all SDHA is associated with sub–100-kDa assemblies (Fig. 2D), we speculate that SDHAF2 accumulation in SDHB^KO^ is due to formation of a nonproductive assembly intermediate as is often seen in mutants affecting the assembly of respiratory chain complexes (*24*, *42*, *43*). Furthermore, while oxygen consumption measurements revealed that inhibition of CV with oligomycin rapidly suppresses mitochondrial respiration (fig. S1G), treatment with oligomycin for 8 hours did not alter SDHAF2 abundance (Fig. 2B). The analysis of the ubiquinol/ubiquinone ratio revealed a reduction in ubiquinone levels following oligomycin treatment compared to control cells (fig. S5A). Together, we propose that in antimycin A–treated HEK293T cells, increased SDHAF2 levels are modulated by changes in the Q-pool redox state to avoid further electron flux into the Q-pool. The mechanism underpinning this is explored below.

### CII assembly drives ROS-mediated adaptation to CIII dysfunction

**Fig. 7.**
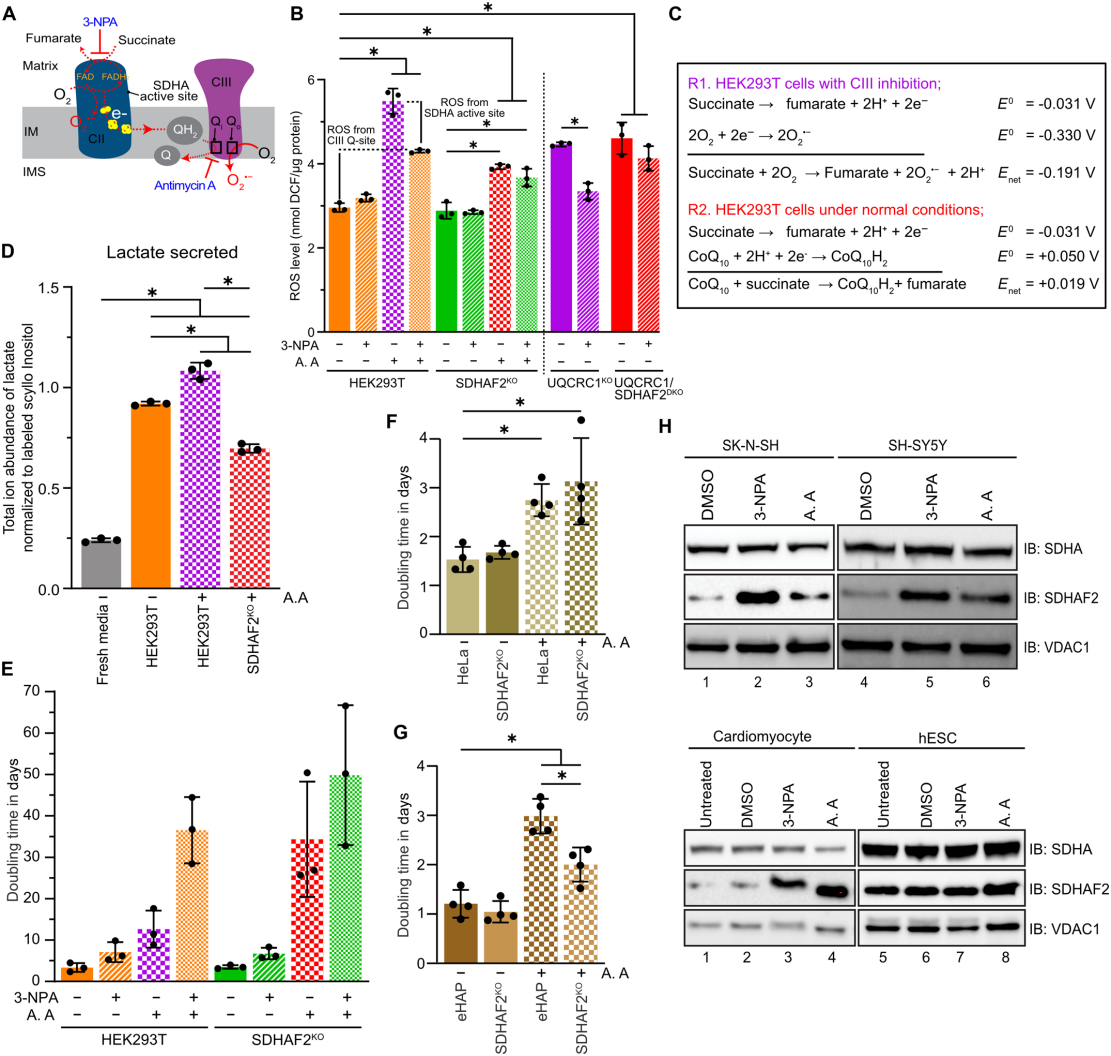
CII assembly drives ROS-mediated adaptation to CIII dysfunction. (**A**) Schematic describing electron flux and sites of ROS generation in complexes II and III. (**B**) Total cellular ROS was measured in the indicated cell lines following either CII or CIII inhibition, or cotreated with both, as indicated. Mean values ± SD, *n* = 3 replicates per condition. *P > 0.05. *P* values were calculated using a one-way ANOVA. (**C**) Possible redox reactions at CII in response to functional and dysfunctional CIII in HEK293T cells and their reduction potentials. Reaction 1 (R1): During CoQ-pool stress, electron flux into oxygen due to increased succinate oxidation. Reaction 2 (R2): Electron flux into CoQ-pool during canonical TCA cycling. (**D**) Measurement of lactic acid secreted in media from the indicated cell lines treated with or without antimycin A. Means ± SD, *n* = 3 replicates. *P > 0.05. *P* values were calculated using a one-way ANOVA. (**E**) Doubling times of control and SDHAF2^KO^ HEK293T cells treated with either CII or CIII inhibitors or cotreated with both, monitored using an Incucyte Live cell imaging system. Means ± SD, *n* = 3. (**F** and **G**) Doubling times of control and SDHAF2^KO^ HeLa and eHAP cells treated with or without CIII inhibitor as described in (E). Means ± SD, *n* = 4. *P > 0.05. *P* values were calculated using a one-way ANOVA. (**H**) Assessment of SDHAF2 response to 3-NPA and antimycin A in SK-N-SH and SH-SY5Y, human H9 embryonic stem cells (hESC), and hESC cells differentiated into cardiomyocytes. Cells were treated with the indicated inhibitors or DMSO for 8 hours. Cell lysates were subjected to SDS-PAGE and immunoblotting using the indicated antibodies. IM, inner membrane; IMS, intermembrane space.

ROS can have both advantageous and detrimental effects on cellular homeostasis. Almost 90% of cellular ROS is generated in mitochondria, primarily from CI and CIII (*44*, *45*). CII only makes a minor contribution to the generation of ROS under normal growth conditions. However CII can be a significant contributor of ROS during OXPHOS dysfunction (*46*, *47*). ROS generation occurs at the active site of SDHA within CII and can be driven by both forward (from succinate) and reverse (from ubiquinol) reactions (*48*, *49*). To investigate the impact of SDHAF2 on ROS generation during OXPHOS dysfunction, we measured cellular ROS following 8 hour treatments with combinations of 3-NPA, which irreversibly blocks the active site of SDHA within CII but does not lead to ROS generation and antimycin A, which binds to the Q_i_ site of CIII, leading to ROS production at the Q_o_ site (Fig. 7A). As expected, increased ROS production occurs in control HEK293T cells following treatment with antimycin A (Fig. 7B). No increase in ROS was observed following 3-NPA treatment alone, consistent with CII being a minor contributor to ROS production under normal growth conditions. Cotreatment of cells with both 3-NPA and antimycin A resulted in a significant reduction in ROS compared to antimycin A treatment alone (Fig. 7B, compare purple and red hatched bars in control HEK293T cells), indicating that CII is a significant source of ROS during CIII dysfunction as succinate oxidation leads to reduction of oxygen and generation superoxide radicals. Note that this is not a thermodynamically favorable reaction (see first set of reactions in Fig. 7C), and under normal conditions, electrons would flow into ubiquinone (see second set of reactions in Fig. 7C); however, due to the accumulated ubiquinol pool (Fig. 6A), only reduction of oxygen becomes possible. While SDHAF2^KO^ cells treated with antimycin A produce ROS (Fig. 7B), they do so only at the level found in control cells cotreated with antimycin A and 3-NPA. Cotreatment of SDHAF2^KO^ with 3-NPA and antimycin A did not further reduce ROS levels (compare antimycin A–treated SDHAF2^KO^ cells with and without 3-NPA treatment), suggesting that the ROS generated in the SDHAF2^KO^ treated with antimycin A arises solely from CIII inhibition and not at the SDHA active site at CII. In this scenario, the large reduction in forward SDH activity (Fig. 5C) from impaired CII assembly in SDHAF2^KO^ leads to an absence of CII-derived ROS (Fig. 7B). Supporting this conclusion is the comparable level of the ubiquinone to ubiquinol ratio in UQCRC1^KO^ or antimycin A–treated control cells irrespective of the presence of SDHAF2 (Fig. 6A) indicating no additional electron flux into the Q-pool through CII.

Because the net reduction potentials (*E*_net_) shows reduction of ubiquinone into ubiquinol is more favorable than reduction of oxygen into superoxide (Fig. 7C), in HEK239T cells, under conditions of impaired OXPHOS fluxing additional electrons into the Q-pool via CII would lead to further Q-pool stress. Instead, we propose that flux of electrons into oxygen at the CII active site, facilitated by an ability to dynamically increase CII assembly and thus activity, facilitates a ROS-mediated metabolic shift toward glycolysis (*50*, *51*). To test this, we quantified the relative abundance of lactic acid in the growth media normalized by cell number from control HEK293T cells with or without antimycin A treatment and antimycin A–treated SDHAF2^KO^ cells using GC-MS and an MRM quantification strategy. Control HEK293T cells treated with antimycin A showed significantly higher levels of lactic acid compared to untreated cells (Fig. 7D). However, media lactic acid was not increased in antimycin A–treated SDHAF2^KO^ cells. We next assessed cell proliferation in the same cell lines cultured in glucose-containing media and treated with either 3-NPA and/or antimycin A. We reasoned that the presence of SDHAF2 would enhance cell proliferation following CIII inhibition. Treatment of control HEK293T cells with antimycin A led to a reduction in growth rate, which was markedly exacerbated in combination with 3-NPA (Fig. 7E and fig. S5B). We observed a similarly severe growth defect in the SDHAF2^KO^ upon cotreatment with 3-NPA and antimycin A. While SDHAF2^KO^ cells treated with antimycin A alone showed a slightly improved growth rate compared to cotreatment with 3-NPA, control HEK293T cells treated with antimycin A (i.e., cells that retain the ability to dynamically increase SDHAF2 levels) had a large improvement in proliferation over similarly treated SDHAF2^KO^ (Fig. 7E, compare purple and red checked bars). This is consistent with a synergetic rather than additive impact on proliferation following SDHAF2 loss (fig. S5B, compare orange circles/dashed lines, with green triangles/dashed lines, quantified in Fig. 7E). The severe growth phenotype of antimycin–A treated SDHAF2^KO^ relative to antimycin A–treated HEK293T cells could be partially rescued by reintroduction of SDHAF2^FLAG^ (fig. S5B, compare green triangles/dashed lines with black circles), although variability in confluence due to continual media changes necessitated by doxycycline use prevented robust doubling time calculations. In contrast, the knockout of SDHAF2 in HeLa cells did not lead to a compounded growth phenotype when treated with antimycin A (Fig. 7F and fig. S5C) unlike HEK293T cells (Fig. 7E and fig. S5B). Similar observations were made in the eHAP background (Fig. 7G), which, similar to HeLa cells, do not respond to antimycin A by accumulating SDHAF2 (Fig. 5F). Last, we asked if other cell types respond to antimycin A treatment by accumulating SDHAF2 as HEK293T cells do. As can be seen in Fig. 7H, neuroblastoma-derived SK-N-SH and SH-SY5Y cells, which are known for the neuronal-like phenotype, tolerate growth in galactose and have spare respiratory capacity (*52*) readily accumulate SDHAF2 following treatment with antimycin A (Fig. 7H, top panels). Likewise, while highly glycolytic H9 human embryonic stem cells (hESCs) (*53*, *54*) do not accumulate SDHAF2 following antimycin A treatment (Fig. 7H, bottom panels); however, the same cells differentiated into cardiomyocytes strongly accumulate SDHAF2. Together, while our results are consistent with reports that different cell types exhibit a varying capacity to support a net reductive TCA cycle during OXPHOS stress (*7*), in cell types not already adapted to glycolysis, this would come at the cost of an impaired ability to use CII-mediated ROS signaling and thus an ability to drive a shift toward glycolytic metabolism. We propose that these cell types, including HEK293T cells, avoid this through dynamically modulating CII assembly.

## DISCUSSION

Human SDHAF2 and its homologs in yeast and bacteria have established roles in promoting the flavination of SDHA. Our data and results from similar studies using gene-edited human cell lines (*14*–*16*) demonstrate that SDHAF2 is not essential for this process or for the activity of CII, which we show is retained in the absence of SDHAF2, although at a 30 to 45% reduced rate (Fig. 1A and fig. S1A). This result is in agreement with recent in vitro studies on purified human proteins that show that flavination of SDHA can occur in the absence of SDHAF2, provided that FAD and a dicarboxylate such as fumarate are present (*2*). In addition to its assembly into mature CII, we (Fig. 2E) and others (*2*) show that SDHA is found in several sub–100-kDa assemblies with SDHAF2 and/or SDHAF4. These assemblies were initially referred to as a single complex known as CII_low_ and were suggested to have a role in the regulation of metabolite synthesis following OXPHOS stress (*4*). Subsequent work from the same groups suggested that while an SDHA-SDHAF2 complex is the predominant assembly under pathological conditions such as OXPHOS dysfunction, during normal conditions, SDHA is passed from SDHAF2 to SDHAF4 upon its flavination to support CII maturation (*2*). Our data are consistent with this model and further show that SDHAF2 associates with a mixture of flavo- and apo-SDHA (Fig. 1E) and that fully assembled CII likely only contains mature flavo-SDHA. We show that the ~55-kDa SDHA-SDHAF2 subassembly accumulates in HEK293T cells following both acute and chronic OXPHOS dysfunction (Fig. 2D), and its absence during OXPHOS dysfunction results in a net reductive TCA cycle (Fig. 5D) with catastrophic consequences to cellular proliferation (Fig. 3C) due to lack of a ROS-mediated switch to glycolytic metabolism (Fig. 7, B and D). Moreover, the accumulation of SDHAF2 following OXPHOS insult is notably absent in some cell lines (HeLa, eHAP, 143B, MCF7, undifferentiated H9 hESCs) but not others (HEK293T, SK-N-SH, SH-SY5Y neuroblastoma cell lines, and hESC-differentiated cardiomyocytes) (Figs. 5F and 7I). In cells where accumulation of SDHAF2 does not occur, a net reductive TCA cycle is well tolerated and readily proliferate with or without SDHAF2 being present (Fig. 7, F and G).

Mitochondrial metabolic reprogramming commonly occurs during inflammation, metastasis, and ischemic hypoxia. In particular, the inhibition of respiratory chain complexes leads to changes in metabolic flux needed to maintain the bioenergetic and redox balance of cells and promote cell proliferation and survival (*50*). It is well established that cells initiate HIF-1α–driven metabolic reprogramming under various pathological conditions, wherein succinate accumulation via CII inhibition plays a critical role in stabilizing HIF-1α. Our findings revealed almost indistinguishable levels of succinate accumulation in HEK293T cells with dysfunctional CIII, regardless of the presence or absence of SDHAF2 (Fig. 4F, compare purple and red bars). Further, the improved proliferation rate (Fig. 3C) in HEK293T cells that retain an ability to accumulate SDHAF2 (i.e., control HEK293T cells treated with antimycin A) over the untreated SDHAF2^KO^ cell line is not attributable to changes in TCA cycle directionality, as in the former we observed no net forward or reverse CII activity (Fig. 5D). In contrast, these cells produced significant SDHA active site–derived ROS from CII, another well-known HIF-1α–stabilizing agent (*50*, *55*), which was lost in the absence of an ability to accumulate the SDHAF2-SDHA assembly (Fig. 7B) along with a shift to glycolysis as evidenced by increased media lactic acid (Fig. 7D).

How accumulation of the SDHAF2-SDHA assembly mediates this phenomenon in HEK293T and presumably other cell types similarly responding to OXPHOS stress is not fully clear. However, noting the very low basal amount of SDHAF2-SDHA in HEK293T cells compared to the nonresponsive HeLa and eHAP cell lines, we propose a model that underpins a need of some cell lines to dynamically increase CII assembly and maintain ROS-mediated glycolytic signaling during acute OXPHOS stress. In contrast, cell lines that are adapted to glycolysis, such as HeLa cells that grow poorly in galactose compared to HEK293T cells (fig. S1H), have lower basal OCR and spare respiratory capacity (Fig. 1G) and lower basal CII levels (Fig. 1A), can tolerate a ~30 to 45% reduction in SDHA flavination in the SDHAF2^KO^ and thus a reduced ability to drive CII assembly, given the lower level of CII and lack of a need for ROS-mediated metabolic reprogramming.

It is important to acknowledge that a major limitation of our study is the use of a small number of cell culture–adapted cell lines for the experiments, most of which are tumor derived. It will be critical to establish if this behavior exists in vivo, for example, in mouse tissues where Spinelli *et al.* (*7*) previously demonstrated varying degrees of succinate oxidation and fumarate reduction. Nevertheless, SDHAF2 accumulation readily occurs in hESC-derived cardiomyocytes but not undifferentiated hESCs (Fig. 7H), which are known to favor glycolysis (*53*, *54*), suggesting an acute ability to modulate CII assembly is not restricted to HEK293T cells. Despite having a presumed kidney origin, HEK293T cells have been shown to exhibit neuronal characteristics (*56*). This makes it tempting to extrapolate a similar mechanism being present in neuroblastoma-derived SK-N-SH and SH-SY5Y cells that both accumulate SDHAF2 in response to antimycin A treatment (Fig. 7H) and have other metabolic characteristics of OXPHOS-adapted cells such as spare respiratory capacity (*57*).

Together, our study shows that accumulation of SDHAF2 upon acute OXPHOS dysfunction stabilizes SDHA in the previously observed ~55-kDa SDHAF2-SDHA assembly in some cell lines, with important impact on metabolic adaptation that we suggest is required in cell types with a reliance on mitochondrial OXPHOS. Thus, our study highlights how mitochondrial respiratory chain complex assembly can dynamically adapt to the wider metabolic requirements of the cell.

## MATERIALS AND METHODS

### Routine cell culture and proliferation assays

HEK293T, HeLa, 143B, SK-N-SH, and SH-SY5Y cell lines were cultured in Dulbecco’s modified Eagle’s medium (DMEM; Thermo Fisher Scientific) supplemented with 10% (v/v) fetal bovine serum (FBS; CellSera), uridine (50 μg/ml; Sigma-Aldrich), and a mixture of streptomycin (100 μg/ml) and penicillin (100 U/ml; Thermo Fisher Scientific). eHAP cells were cultured in Iscove’s modified Dulbecco’s medium (Thermo Fisher Scientific) supplemented with 10% (v/v) FBS, uridine (50 μg/ml), and a mixture of streptomycin (100 μg/ml) and penicillin (100 U/ml). For assays involving the inhibition of OXPHOS, inhibitors were used in the following concentrations: 1 μM rotenone (Sigma-Aldrich), 5 mM 3-nitropropionic acid (3-NPA) (Sigma-Aldrich), 30 μM antimycin A (Sigma-Aldrich), 5 mM sodium azide (Fisher Chemical), and 1 μM oligomycin (Sigma-Aldrich). For hypoxic treatment, medium was pretreated in a hypoxia incubator chamber (STEMCELL Technologies) with atmosphere purged by a gas mixture containing 5% CO_2_, 1% O_2_, and 94% N_2_ for 10 min at a flow rate of 25 liter/min. Cells passaged into the pretreated media were placed in the chamber, which was purged as above, following which the chamber was sealed and incubated for 24 hours at 37°C.

To compare growth rates, cells were seeded at a density of 10,000 cells per well in a standard 12-well tissue culture dish unless otherwise mentioned. Cellular proliferation was monitored in real time by imaging at 4-hour intervals over a period of 6 days using an Incucyte imaging system (Sartorius). Doubling time was defined during the logarithmic growth phase using the equation ln(2)/growth rate.

### Stem cell culture and cardiac differentiation

The H9 (WAe009-A) hESC line (*58*) was cultured following a standard protocol as previously described (*59*, *60*). Briefly, the cells were regularly passaged on mitotically irradiated murine embryonic fibroblasts (MEFs) serving as a feeder layer. The culture medium used was serum-free induced pluripotent stem cell (iPSC) media supplemented with basic fibroblast growth factor (50 ng/ml), and the medium was changed daily. Stem cells were then transferred onto Matrigel-coated dishes (In Vitro Technologies) for three consecutive passages to remove the MEFs before drug treatment.

Differentiation into cardiomyocytes was achieved through a small-molecule Wnt activation/inhibition approach in a monolayer culture system, with slight modifications to a previously described protocol (*61*). In brief, H9 hESCs were passaged onto Matrigel-coated dishes at ~25% confluence, 2 days before the differentiation process (day −2). iPSC medium was refreshed on the following day. On day 0, the medium was changed to basal cardiac differentiation medium, composed of RPMI 1640 containing 2% B-27 without vitamin A, 1% GlutaMAX, and 0.5% penicillin-streptomycin, further supplemented with activin A (80 ng/ml; R&D Systems), 12 mM CHIR 99021 (Tocris), and ascorbic acid (50 μg/ml; Sigma-Aldrich) to induce the differentiation. After 24 hours (day 1), the medium was replaced with fresh basal differentiation medium containing 5 mM IWR-1 (Sigma-Aldrich) and ascorbic acid (50 μg/ml) and repeated on days 2, 3, and 5 of differentiation. On day 7, cells were maintained in basal differentiation medium, and fresh medium was changed on day 9. The cells were then cryopreserved on day 11 of differentiation. Cryopreserved cardiomyocytes were thawed in basal differentiation medium containing the ROCK1 inhibitor Y-27632 (In Vitro Technologies). Twenty-four hours later, medium was replaced with basal differentiation medium without Y-27632. To enrich myocyte populations, cardiac differentiations were treated for 72 h with glucose-free RPMI (Thermo Fisher Scientific) supplemented with 5 mM sodium l-lactate, 1% GlutaMAX, and 0.5% penicillin-streptomycin, with medium exchange every 24 hours (*62*). Enriched cardiomyocytes were then passaged on Matrigel-coated dishes in alpha minimum essential medium (αMEM) base media (Thermo Fisher Scientific) supplemented with 10% FBS, 0.5% penicillin-streptomycin, ascorbic acid (50 μg/ml) and Y-27632. The αMEM base medium was refreshed the following day to remove Y-27632 before drug treatment.

### Gene editing and expression

HEK293T knockouts for NDUFA8 (*24*), UQCRC1 (*23*), and COX4 (*63*) were published previously. Guide RNAs targeting SDHB (5′-C- GGTTGCCGGCCACAACCCT), SDHAF2 (5′-AGTGTTCTCGACTTCGTCGC), and ATP5PD (5′-GCCTTTCCTTGTGGGCAGGT) were designed using the CHOPCHOP tool (*64*) and subcloned into pSpCas9(BB)-2A-GFP (green fluorescent protein) (PX458) (*65*) (Addgene plasmid no. 48138) as previously described (*24*). The plasmids were validated by sequencing and then transfected into target cell lines using Lipofectamine 3000 Transfection Reagent (Thermo Fisher Scientific) according to manufacturer’s guidelines. Single cells expressing GFP were isolated on a FACSAria Fusion (BD Biosciences) cell sorter, and clonal populations were subsequently screened for relevant gene knockouts using SDS-PAGE and immunoblotting. For complementation of knockouts, cDNAs encoding SDHB^FLAG^ and SDHAF2^FLAG^ were cloned into pBMN-puro (modified from pBMN-Z, Addgene plasmid no. 1734) and pLVX-TetOne-Puro (Clontech), respectively. The sequence-confirmed constructs were introduced into the respective knockout cells using retroviral and lentiviral delivery systems, respectively, as previously described (*24*, *25*). Different levels of SDHAF2 expression were achieved through doxycycline titration, as shown in Fig. 2F. For expression of the FLAG-tagged AOX, we used the plasmid pCW57.1_AOX-FLAG (Addgene plasmid no. 177984). The transduction was performed using a lentiviral delivery system. Cells integrating the cassette were selected by treatment with puromycin (1 μg/ml; Sigma-Aldrich) for 72 hours. The expression of AOX was achieved by the addition of doxycycline (150 ng/ml).

### Mitochondrial isolation

Mitochondria were isolated using differential centrifugation following a previously described method (*66*). Briefly, cells at confluency were harvested using trypsin and washed with PBS. Cell pellets were then homogenized in isolation buffer (20 mM Hepes-KOH (pH 7.4), 220 mM mannitol, 70 mM sucrose, 1 mM EDTA, 0.5 mM phenylmethylsulfonyl fluoride (PMSF), and bovine serum albumin (BSA) (2 mg/ml) using an ice-cold glass homogenizer (VWR). The homogenates were centrifuged at 800*g* for 5 min at 4°C, and the supernatant was removed and again centrifuged at 10,000*g* for 10 min at 4°C. Pellets containing crude mitochondria were washed with isolation buffer [without BSA (2 mg/ml) followed by centrifugation as above. The pellets were then resuspended in storage buffer [500 mM sucrose and 10 mM Hepes-KOH (pH 7.4)], and protein concentrations were determined by bicinchoninic acid assay (BCA; Thermo Fisher Scientific) before storing at −80°C until further use.

### Gel electrophoresis and immunoblotting

For SDS-PAGE analysis, cell pellets were resuspended into NP-40 lysis buffer containing 50 mM tris-HCl, 150 mM NaCl, 1 mM EDTA, and 1% NP-40 (adjusted to pH 8.0) for 20 minutes. The lysate was clarified by centrifugation at 5,000*g* for 5 min. Protein concentration was determined using the BCA assay and 30 μg of lysate combined with lithium dodecyl sulfate sample buffer (Invitrogen) containing 10 mM dithiothreitol. Samples were resolved using Bolt bis-tris gels (4 to 12%) (Invitrogen) following the manufacturer’s instructions. Samples from cells subjected to hypoxia or CoCl_2_ treatment were solubilized in NP-40 lysis buffer supplemented with 1 mM PMSF and 1x cOmplete protease inhibitor cocktail (Merck) and processed on ice until loading. BN-PAGE was performed as previously described (*21*, *67*). A 30 μg of isolated mitochondria was resuspended in 1 x solubilization buffer [20 mM tris-Cl (pH 7.4), 50 mM NaCl, 0.1 mM EDTA, 10% (v/v) glycerol, and 1% (w/v) digitonin] and incubated on ice for 30 min. The samples were clarified by centrifugation at 20,000*g* for 10 min at 4°C, and 10× BN-PAGE loading dye containing 5% (w/v) Coomassie Brilliant Blue G-250 (Thermo Fisher Scientific), 500 mM ε-amino-*n*-caproic acid (Sigma-Aldrich), and 160 mM bis-tris (pH 7.0) was added before loading on Invitrogen NativePAGE bis-tris gels (3 to 12%). For 2D-PAGE and complexome, in-house BN-PAGE gradient gels were prepared as previously described (*68*). Briefly, 40% (w/v) acrylamide stock solution [acrylamide/bisacrylamide (37.5:1)] was diluted in blue native gel buffer (66 mM ε-amino-*n*-caproic acid and 50 mM bis-tris (pH 7.0)] to prepare 4, 13, and 16% gradient gel mixtures, with 13% glycerol addition to the 13 and 16% mixtures. The 4 to 13% or 4 to 16% gradient separation gels were poured using a gradient mixer. To analyze flavinated SDHA in mature CII by 2D-PAGE, 100 μg of isolated mitochondria was solubilized with 1x solubilization buffer and resolved using the above 4 to 13% BN-PAGE gradient gel. The relevant lanes were excised and polymerized in the stacker of an in-house tris-tricine gradient gel (10 to 16%), as previously described (*21*, *69*).

Electrophoresis buffers and running conditions were used as per the manufacturer’s instructions. Following electrophoresis, proteins were transferred onto PVDF membranes with a 0.45-μm pore size (Millipore) using a semi-dry transfer apparatus (Thermo Fisher Scientific) and subjected to immunoblot analysis using the following antibodies: SDH5/SDHAF2 (Cell Signaling Technology, #45849S), SDHA (Abcam, #ab14715), SDHB (Abcam, #ab14714), UQCRC1 (Thermo Fisher Scientific, #16D10AD9AH5), FLAG (Sigma-Aldrich, #F1804), total OXPHOS Blue Native WB Antibody Cocktail (Abcam, #ab110412), ATP5A (Abcam, #ab14748), TIMM23 (BD Biosciences, #611222), and HIF-1α (Santa Cruz Biotechnology, #SC-13515). In-house rabbit antibodies (*70*) were used for the detection of VDAC1 and mtHsp-70. Horseradish peroxidase–linked anti-mouse immunoglobulin(IgG) and anti-rabbit IgG (Cell Signaling Technology) were used as secondary antibodies, and blots were imaged using Clarity Western ECL substrate (Bio-Rad) and a BioRad ChemiDoc imaging system.

### SDHA flavination assays

The flavination of SDHA was determined using a previously described ultraviolet-transilluminator–based method (*9*, *12*, *14*). Proteins from isolated mitochondria were resolved by SDS-PAGE as described above. The gel was treated with 10% acetic acid for 20 min, and fluorescence emission from covalently bound flavin (FAD) upon excitation at 488 nm was imaged using a Bio-Rad ChemiDoc imaging system. For IPMS experiments, control and FLAG-tagged SDHAF2 and SDHB cells were harvested in triplicates and then solubilized in a buffer containing 20 mM tris-Cl (pH 7.4), 50 mM NaCl, 10% (v/v) glycerol, 0.1 mM EDTA, 1% (w/v) digitonin, and benzonase, followed by incubation on ice for half an hour. The lysates are then centrifuged at high speed for 5 min at 4°C. Spin Columns (Pierce Thermo Fisher Scientific) were packed with 40 μl of FLAG beads slurry (Sigma-Aldrich) and equilibrated using a wash buffer composed of 20 mM tris-Cl (pH 7.4), 60 mM NaCl, 10% (v/v) glycerol, 0.5 mM EDTA, and 0.1% (w/v) digitonin. The lysates were then loaded to the FLAG-affinity columns and incubated for 2 hours at 4°C with gentle rotation. After the incubation, columns were spun briefly at 300*g* to bring the beads down to the bottom of column. Next, columns were washed with 500 μl of wash buffer 20 times. A 50 μl of wash buffer containing FLAG peptide (100 μg/ml; Sigma-Aldrich) was then added to the column, followed by incubation for 30 min at 4°C. The columns were then centrifuged, and eluted proteins were collected. This was followed by a second elution with 50 μl of wash buffer (without FLAG peptide). Both eluates were then pooled together.

Proteins were then digested using a S-trap Micro Spin Columns (ProtiFi) according to the manufacturer’s instructions. Briefly, lysis buffer containing SDS and triethylammonium bicarbonate was added to the eluate to denature proteins. Proteins were further reduced and alkylated using 10 mM tris(2-carboxyethyl)phosphine hydrochloride (TCEP; Bond-Breaker, Thermo Fisher Scientific) and 40 mM chloroacetamide (Sigma-Aldrich). Proteins were digested using trypsin as 1:20 trypsin to protein ratio at 37°C overnight. The peptides were then eluted and dried using a CentriVap Benchtop Vacuum Concentrator (Labconco). The peptides were reconstituted in 0.1% trifluoroacetic acid (TFA) and 2% ACN and analyzed by LC-MS/MS on an Orbitrap Ascend Mass Spectrometer (Thermo Fischer Scientific) over a 95-min gradient. Tryptic peptides were loaded onto an Acclaim Pepmap nano-trap column (Dinoex-C18, 100 Å, 75 μm by 2 cm) at an isocratic flow of 5 μl/min of 2% acetonitrile (ACN) and 0.1% formic acid (FA) for 6 min before switching with an Acclaim Pepmap RSLC analytical column (Dinoex-C18, 100 Å, 75 μm by 50 cm). The separation was performed using a nonlinear 95-min gradient of solvent A [5% dimethyl sulphoxide (DMSO) and 0.1% FA in water) and solvent B (5% DMSO, 100% ACN, and 0.1% FA). The flow gradient was (i) 0 to 6 min at 3% solvent B, (ii) 6 to 7 min at 4% solvent B, (iii) 7 to 82 min 25% solvent B, (iv) 82 to 86 min at 40% solvent B, (v) 86 to 90 min at 80% solvent B, and (vi) 90 to 95 min at 3% solvent B and equilibrated at 3% solvent B for 10 minutes before the next injection. The data were acquired using positive polarity with a MS1 scan range of 300 to 1350 mass/charge ratio (*m*/*z*). The resolution was set to 120,000, ACG target of 1 × 10^6^, and maximum injection time was set to 250 ms. Mass spectrometer was operated in the data-dependent mode with a targeted inclusion list containing unmodified histidine and FAD-modified histidine for SDHA peptide (SHTVAAQGGINAALGNMEEDNWR). The isolation window was set to 1.2 *m*/*z*. Precursors were fragmented with a normalized collision energy of 25%. The resolution was set to 15,000, automatic gain control (AGC) target was set to 1.5 × 10^5^, with a maximum injection time of 27 ms.

Raw files were searched against the UniProt human database (June 2024) using MaxQuant (version 2.5.2.0) (*71*). Trypsin/P was specified as the digestion enzyme with up to three missed cleavages. The parameters were carbamidomethylation on cysteine as fixed modification, oxidation of methionine, and acetylation of protein N terminus as variable modification along with FAD (783.1414) on histidine. Peptide and protein false discovery rate (FDR) were set to 0.05. Data analysis was done on Perseus (version 1.6.10.43) (*72*). Proteins not quantified in all bait replicates were removed. The missing values in control were imputed, and a *t* test with permutation-based FDR (*S*_0_ = 1) used to identify significantly enriched proteins.

### Respiratory chain enzymology

The activity of CI, CII, and citrate synthase (CS) were measured using spectrophotometric enzyme assays of enriched mitochondrial fractions prepared from cell pellets following previously described methods (*73*). For the measurement of rotenone-sensitive CI activity, reduced form nicotinamide adenine dinucleotide (NADH) oxidation was monitored at 340 nm in assay buffer containing 50 mM KPi (pH 7.4), BSA (1 mg/ml), 50 μM NADH, 10 μM antimycin A, 1 mM KCN, and 50 μM ubiquinone, with or without 2.5 μM rotenone. For the CII activity assay, ubiquinone reduction was monitored at 280 nm in assay buffer containing 50 mM KPi (pH 7.4), 10 mM sodium succinate, 2.5 μM rotenone, 10 μM antimycin A, 1 mM KCN, and 50 μM ubiquinone_1_. CS activity was assessed by monitoring the formation of 5-thio-2 nitrobenzoate anions at 412 nm in assay buffer containing 50 mM KPi (pH 7.4) and 0.1 mM 5,5′-dithio-bis-(2-nitobenzoic acid) (DTNB). This indirect measurement of CS activity results from the spontaneous reaction of the thiol reagent DTNB with the free sulfhydryl groups of coenzyme A (CoA.SH), resulting from the condensation of acetyl-CoA with oxaloacetate to form citrate. Following enzyme activity measurements, the sample protein concentration was quantified using a BCA assay, and enzyme activity was calculated.

### Oxygen consumption

HEK293T and HeLa cells were seeded at densities of 25,000 and 15,000 cells per well, respectively, on a Seahorse assay microplate (Agilent) the day before the experiment. On the day, the growth medium was replaced with the serum-free XF assay medium (Agilent) supplemented with 10 mM glucose, 2 mM glutamine, and 1 mM pyruvate. The cells were then incubated in a non-CO_2_ incubator at 37°C. OCRs were measured using a Seahorse XFe96 Analyzer (Agilent) with measurement cycles of 3-min mix and 3-min measure, following sequential addition of oligomycin (2 μM), FCCP (0.5 μM), and rotenone + antimycin A (0.5 μM each), according to the manufacturer’s protocol. OCR measurements were normalized after the experiment based on the cell number per well calculated using CyQuant (Invitrogen).

### SILAC labeling and whole-cell quantitative proteomics

SILAC labeling and quantitative proteomics were conducted as described in our previous studies (*24*). Briefly, SILAC medium (“light” and “heavy”) was prepared by supplementing arginine- and lysine-free DMEM as above with 10% (v/v) dialyzed FBS (Thermo Fisher Scientific); l-proline; and either light or heavy ^13^C_6_,^15^N_2_-lysine and ^13^C_6_,^15^N_4_-arginine (Silantes). Control and knockout HEK293T cells labeled in triplicate with a label switch were solubilized in 1% (w/v) sodium deoxycholate and 100 mM tris-HCl (pH 8.1). Lysates were alkylated with 5 mM TCEP, 20 mM chloroacetamide and digested with trypsin overnight at 37°C. Detergent was removed by ethyl acetate extraction in the presence of 2% FA, and peptides were concentrated by vacuum centrifugation. Peptides were reconstituted in 0.5% FA and loaded onto small cation exchange (Empore Cation Exchange-SR, Supelco Analytical) stage-tips. Tips were washed with 20% ACN and 0.5% FA and eluted in five fractions of increasing (45 to 300 mM) ammonium acetate, 20% ACN and 0.5% FA. A sixth elution was collected using 5% ammonium hydroxide and 80% ACN. Fractions were desalted using in-house SDB-XC stage-tips (Empore SDB-XC, Supelco Analytical) as described previously (*23*). Peptides were reconstituted in 0.1% TFA and 2% ACN and analyzed by online nano–high-performance LC (HPLC)/electrospray ionization-MS/MS on a Q Exactive Plus connected to an Ultimate 3000 HPLC (Thermo Fisher Scientific). Peptides were loaded onto a trap (Acclaim C18 PepMap nano Trap × 2 cm, 100-μm inner diameter, 5-μm particle size, and 300-Å pore size; Thermo Fisher Scientific) before switching the trap in line with the analytical column (Acclaim RSLC C18 PepMap Acclaim RSLC nanocolumn, 75 μm by 50 cm, PepMap100 C18, 3-μm particle size, 100-Å pore size; Thermo Fisher Scientific). The separation of peptides used a custom nonlinear gradient of buffer A (0.1% FA and 2% ACN) and buffer B (0.1% FA and 80% ACN) over 120 min. Data were collected in positive mode using data-dependent acquisition and the same instrument parameters as described (*24*). Raw files were processed using MaxQuant (version 1.6.12.0) (*71*) against the UniProt human database including reviewed, canonical, and isoform entries (March 2020). Default settings for a multifraction SILAC-multiplexed analysis were used with the exception of enabling “LFQ” “requant” and “Match between runs”. The output was loaded into Perseus (version 1.6.10.43) (*72*), and identifications were matched to MitoCarta3.0 annotations (*74*). Dataset cleaning comprised removal of proteins annotated as “only identified by site,” “reverse,” “potential contaminant,” and proteins identified from less than two unique peptides. Normalized SILAC ratios were log2-transformed, and a single sample Student’s *t* test was performed on replicates for each knockout.

### Complexome proteomics

For complexome analysis, UQCRC1^KO^ and control HEK293T cells were SILAC-labeled, and crude mitochondria were isolated as described above. Complexome BN-PAGE was performed exactly as described (*23*). Briefly, equal amounts of mitochondria (50 μg) from heavy- and light-labeled cells were combined and solubilized in a standard BN-PAGE buffer containing 1% (w/v) digitonin. Complexes were separated using an in-house BN-PAGE gradient gel (4 to 16%), after which the gel was fixed [50% (v/v) methanol, 10% (v/v) acetic acid, and 10 mM ammonium acetate] for 30 min, followed by a 30-min staining with Coomassie solution [0.025% (w/v) Coomassie and 10% (v/v) acetic acid]. The gel was partly destained in 10% acetic acid and washed in Milli-Q water, and each lane containing the pairs of control and knockout SILAC-labeled mitochondria was excised into 60 even slices. Slices were placed into individual wells of an Acroprep 30- to 40-μm polypropylene/polyethylene-filtered microtiter plate containing 50 mM ammonium bicarbonate for in-gel tryptic digest as described (*23*, *75*). Digested peptides were desalted on SDB-XC stage-tips as above and reconstituted in 2% (v/v) ACN, 0.1% (v/v) TFA for LC-MS/MS analysis on a Q-Exactive Plus mass spectrometer (Thermo Fisher Scientific). The LC system comprised an Acclaim Pepmap nano-trap column (Dinoex-C18, 100 Å, 75 μm by 2 cm) and Acclaim Pepmap RSLC analytical column (Dinoex-C18, 100 Å, 75 μm by 50 cm). Peptides were loaded on to the trap, which was subsequently switched in-line with the analytical column and eluted with an in-house nonlinear 60-min gradient of buffer A [5% DMSO in 0.1% (v/v) FA] and buffer B (5% DMSO in 100% ACN and 0.1% FA). The mass spectrometer was operated in positive mode using data-dependent acquisition. The following parameters were used: full MS1 spectra were acquired at 70,000 resolution, AGC target of 3 × 10^6^, and maximum injection time (IT) time of 50 ms. Fifteen of the most intense peptide ions with charge states ≥2, and intensity threshold of 4 × 10^4^ was isolated for MS/MS. The isolation window was set at 1.2 *m/z*, and precursors were fragmented using normalized collision energy of 30, 17,500 resolution, AGC target of 5 × 10^4^, and maximum IT time of 50 ms. Dynamic exclusion was set to be 30 s. Raw files were processed using MaxQuant (version 1.6.17.0) (*71*) against a UniProt human, canonical, and isoforms database (August 2020). Default settings for a SILAC-multiplexed analysis were used. The calculation of intensity-based absolute quantification (iBAQ) intensities was enabled with the log fit function disabled. Heavy and light iBAQ intensities were imported into Perseus (version 1.6.15.0) (*72*) and nonmitochondrial proteins (as defined in MitoCarta3.0) were removed. The resulting matrix was loaded into NOVA (version 0.8.0.0) for complexome analysis and visualization. Mass scale calibration was performed by exponential interpolation in NOVA by inputting apparent protein masses selected from table S5 of (*76*). Proteins of interest underwent hierarchical clustering using default settings, with optimized leaf ordering, average linkage, and a Pearson correlation distance function, without normalization. Exported heatmaps with iBAQ values were normalized in Excel by row average for heatmap generation and by maximum intensity for profile plot generation.

### Metabolomics

Cells were seeded at 50% confluency in six-well dishes containing DMEM media (Thermo Fisher Scientific) 24 hours before the experiment. For stable isotopolog tracing of ^13^C_6_-glucose, ^12^C containing medium was replaced with glucose-free DMEM (Thermo Fisher Scientific) supplemented with 25 mM ^13^C_6_-glucose (Merck), 1 mM sodium pyruvate (Thermo Fisher Scientific), 10% FBS, uridine (50 μg/ml), and a mixture of streptomycin (100 μg/ml) and penicillin (100 U/ml). For stable isotopolog tracing of ^13^C_5_-glutamine (Merck), medium was replaced with glutamine-free DMEM (Thermo Fisher Scientific) supplemented with 2 mM ^13^C_5_-glutamine, 1 mM pyruvate (Thermo Fisher Scientific), 10% FBS, uridine (50 μg/ml), and a mixture of streptomycin (100 μg/ml) and penicillin (100 U/ml). After 8 hours of incubation in the tracer media, cells underwent polar metabolite extraction, as described below. In relevant experiments, doxycycline and antimycin A were introduced 24 and 8 hours before the addition of tracer and maintained until the completion of the experiment, respectively. Cells were then washed with PBS and snap-frozen by direct addition of liquid nitrogen. Polar metabolites were extracted by adding 600 μl of HPLC grade methanol:chloroform mixture [9:1; (v/v)], containing internal standards 1.66 μM ^13^C_5_,^15^N-valine and 1.66 μM ^13^C_6_-sorbitol mixture for LC-MS analysis and 1 mM scyllo-inositol for GC-MS analysis. After 10-min incubation on ice, cells were harvested, and lysates were centrifuged at 16,100*g* at 4°C for 5 min. For GC-MS, supernatants were dried using a CentriVap concentrator (Labconco) or for LC-MS a stream of N_2_. Dried samples were subsequently subjected to LC-MS analysis for glucose and derivatization, followed by GC-MS for glutamine isotopolog distributions, as described below.

For lactic acid measurement in media, a monophasic extraction protocol was used. To 30 μl of media, 90 μl of 100% MeOH containing 1.66 μM ^13^C_5_,^15^N valine and 1.66 μM ^13^C_6_ sorbitol was added. Each sample was vortexed and then incubated at 4°C for 10 min with continuous agitation (12*g*). The samples were centrifuged at 4°C for 10 min at 16,000*g*. The supernatant was transferred into a fresh 1.5-ml Eppendorf tube, and the precipitate was discarded. A 10-μl aliquot of each sample was pooled to create the pooled biological quality control (PBQC). A 10 μl of each study sample and the PBQC were transferred into HPLC inserts and evaporated at 30°C to complete dryness using a CHRIST RVC 2-33 CD plus speed vacuum before the GC-MS analysis.

For steady-state metabolite profiling, polar metabolites were extracted from snap-frozen cells using 600 μl of HPLC grade methanol:chloroform mixture [9:1; (v/v)], along with internal standards (1.66 μM ^13^C_5_,^15^N-valine and 1.66 μM ^13^C_6_-sorbitol), as described above. The clarified supernatants and PBQCs were dried using a CentriVap concentrator (Labconco). Dried samples were derivatized before GC-MS analysis as described below.

Sample derivatization for GC-MS analysis was carried out online using the Shimadzu AOC6000 autosampler robot, following the manufacturer’s instructions (Shimadzu). Briefly, 25 μl of methoxyamine hydrochloride (30 mg/ml in pyridine, Merck) was added to the samples, and the mixture was shaken at 37°C for 2 hours. Subsequently, A 25 μl of *N*,*O*-bis(trimethylsilyl)trifluoroacetamide with trimethylchlorosilane (BSTFA with 1% TMCS; Thermo Fisher Scientific) was added, and the samples were further derivatized at 37°C for 1 hour. After derivatization, samples were allowed to equilibrate at room temperature for 1 hour before injecting onto the GC column. The GC-MS system used in this study consisted of a 2030 Shimadzu gas chromatograph coupled with a TQ8050NX triple quadrupole mass spectrometer (Shimadzu), with an electron ionization source (−70 eV). The mass spectrometer was tuned following the manufacturer’s guidelines using tris-(perfluorobutyl)-amine (CF43). GC-MS was performed on a 30-m Agilent DB-5 column with a diameter of 0.25 mm and a film thickness of 1 μm. A 1 μl of sample was injected in split mode (1:10) using a hot needle technique. The injection temperature at the inlet was set at 280°C, the MS transfer line at 280°C, and the ion source adjusted to 200°C. Helium was used as a carrier gas with a flow rate of 1 ml/min, and argon gas was used in the collision cell to generate the MRM product ion. The analysis of trimethylsilyl samples was carried out using the following GC oven temperature program: a start temperature of 100°C, held for 4 min, followed by a ramp of 10°C/min until reaching 320°C, and then maintained at 320°C for 11 min. Approximately 520 targets were acquired using the Shimadzu Smart Metabolite Database, with each target having a quantifier MRM and a qualifier MRM, enabling the coverage of approximately 350 endogenous metabolites and multiple stable isotopically labeled internal standards. The resulting data were further processed using Shimadzu LabSolutions Insight software, where peak integrations were visually validated and manually corrected where required. The dataset was then normalized by median and log-transformed. Volcano plots were generated using Perseus v1.6.10.43 (*72*).

For stable isotopolog tracing of ^13^C_5_-glutamine analysis, the mass spectrometer was operated as above. The resultant data were processed using the Shimadzu LabSolutions Insight software where peak isotopolog peaks were visually validated and manually corrected where required. The natural isotopic background correction, fractional labeling, and isotopolog distribution were calculated using the Nanchen method (*77*).

For LC-MS of ^13^C_6_-glucose tracer studies, dried extracts were reconstituted in 75 μl of a mixture of ACN:water [4:1; (v/v)] before analysis. Polar metabolite separation was performed on a Vanquish Horizon UHPLC system coupled to an Orbitrap ID-X Tribrid mass spectrometer (Thermo Fisher Scientific), as described previously (*78*). Briefly, the separation was performed on Merck SeQuant ZIC-pHILIC column (150 mm by 4.6 mm, 5-μm particle size), which was maintained at 25°C. The chromatographic elution involved a binary gradient consisting of solvent A [20 mM ammonium carbonate (pH 9.0, Sigma-Aldrich)] and solvent B (100% ACN). The gradient run was as follows: time (*t*) = 0.0 min, 80% B; *t* = 0.5 min, 80% B; *t* = 15.5 min, 50% B; *t* = 17.5 min, 30% B; *t* = 18.5 min, 5%; *t* = 21.0 min, 5% B; *t* = 23 to 33 min, 80% at a solvent flow rate of 300 μl/min. The mass spectrometer was coupled to a heated electrospray ionization source, and the following conditions were set: sheath gas flow of 40 arbitrary units (Arb), auxiliary gas flow of 10 Arb, sweep gas flow of 1 Arb, ion transfer tube temperature of 275°C, and vaporizer temperature of 320°C. The RF (radio frequency) lens value was 35%. Data were acquired in negative polarity mode with spray voltages of 3500 V. Data analysis was performed using TraceFinder v4.1 (Thermo Fisher Scientific) and El-Maven (www.elucidata.io/el-maven). Level 1 metabolite identification followed the guidelines of the Metabolite Standard Initiative (*79*), where the mass and retention time were matched to the 550 authentic standards available in the Metabolomics Australia in-house library. The natural background correction was performed using the labeled LC-MS/MS workflow tool on the Polly Elucidata platform (*80**,* *81*).

### Steady-state measurement of ubiquinol/ubiquinone ratio

The extraction and relative quantification of ubiquinol and ubiquinone were conducted as previously described (*7*, *41*) with the following modifications. Cell pellets containing 100 μg of protein were vortexed with 250 μl of ice-cold acidified methanol for 10 min at 4°C. Subsequently, 500 μl of ice-cold hexane was added and vortexed for an additional 10 min at 4°C. To separate the CoQ-containing phase, cell lysates were centrifuged at 17,000*g* for 10 min at 4°C. The top hexane layer, containing CoQ, was transferred to a fresh tube and dried using a CentriVap concentrator (Labconco). Dried extracts were reconstituted in a 50-μl mixture of methanol:chloroform [9:1; (v/v)] and subjected to LC-MS analysis using a Vanquish UHPLC connected to an Orbitrap Fusion Lumos mass spectrometer (Thermo Fisher Scientific). Samples were analyzed in separate runs using positive or negative ion modes. Solvent A was 6:4 ACN:water (v/v) with 5 μM medronic acid, and solvent B was 9:1 isopropanol:ACN (v/v). Both solvents contained 10 mM ammonium acetate. A 10 μl of each sample was injected into an Acquity UPLC HSS T3 C18 column (1 mm by 150 mm, 1.8 μm; Waters) at 50°C at a flow rate of 60 μl/min for 3 min using 3% solvent B. During separation, the percentage of solvent B was increased from 3 to 70% in 5 min and from 70 to 99% in 16 min. Subsequently, the percentage of solvent B was maintained at 99% for 3 min. Last, the percentage of solvent B was decreased to 3% in 0.1 min and maintained for 3.9 min. Targeted MS experiments were performed using an electrospray ionization source in positive mode at 3.5 kV. The flow rates of sheath, auxiliary, and sweep gases were 25, 5, and 0 Arb(s), respectively. The ion transfer tube and vaporizer temperatures were maintained at 300° and 150°C, respectively, and the ion funnel RF level was set at 50%. Each full scan MS spectrum was acquired first in the Orbitrap at a mass resolving power of 120,000 (at *m/z* 200) across an *m*/*z* range of 300 to 2000 using quadrupole isolation, an AGC target of 4 × 10^5^, and a maximum injection time of 50 ms. We then targeted higher-energy collisional dissociation (HCD)–MS/MS for ubiquinone at *m/z* 880.7133 and ubiquinol at *m/z* 882.7290 at a mass resolving power of 15,000 (at *m/z* 200), a normalized collision energy (NCE) of 27%, an *m/z* isolation window of 1, a maximum injection time of 35 ms, and an AGC target of 5 × 10^4^. The following transitions were used for the quantification of ubiquinone and ubiquinol: CoQ_10_, 880.7133 > 197.0804; CoQ_10_H_2_, 882.7290 > 197.0804. The peak areas for CoQ_10_ and CoQ_10_H_2_ were extracted using Skyline v22.2, enabling the determination ratio between ubiquinone and ubiquinol.

### ROS measurement

The quantification of H_2_O_2_ was conducted using the OxiSelect in vitro ROS/RNS assay kit according to the manufacturer’s guidelines. Briefly, control HEK293T and knockout cells, treated with or without 3-NPA and antimycin A as above for 8 hours, were lysed using 1% ice-cold Triton X-100 and subsequently centrifuged at 10,000*g* for 5 min at 4°C. A 20-μl aliquot of the resulting supernatant was combined with an equal volume of catalyst in a 96-well opaque-walled plate and incubated for 5 min at room temperature. Following this, 50 μl of detection solution containing 2′,7′-dichlorodihydrofluorescin diacetate probe was added to each well and incubated for 45 min in the dark. The relative fluorescence signal was determined using a CLARIOstar microplate reader (BMG Labtech).

## Supplementary Materials

### The PDF file includes:

Figs. S1 to S5 Legends for datasets S1 to S3

### Other Supplementary Material for this manuscript includes the following:

Datasets S1 to S3
