# Supplementary material for: Complex II assembly drives metabolic adaptation to OXPHOS dysfunction
Source: Sci Adv. 2025 Aug 15;11(33):eadr6012. doi: 10.1126/sciadv.adr6012 (PMC12356253; doi:10.1126/sciadv.adr6012)
Supplement: Supplementary file 1 — Figs. S1 to S5 Legends for datasets S1 to S3 [file sciadv.adr6012_sm.pdf]

Science Advances
AAAS

Supplementary Materials for

Complex II assembly drives metabolic adaptation to OXPHOS dysfunction

Roopasingam Kugapreethan *et al.*

Corresponding author: David A. Stroud, david.stroud@unimelb.edu.au

Sci. Adv. **11**, eadr6012 (2025)
DOI: 10.1126/sciadv.adr6012

The PDF file includes:

Figs. S1 to S5
Legends for datasets S1 to S3

Other Supplementary Material for this manuscript includes the following:

Datasets S1 to S3

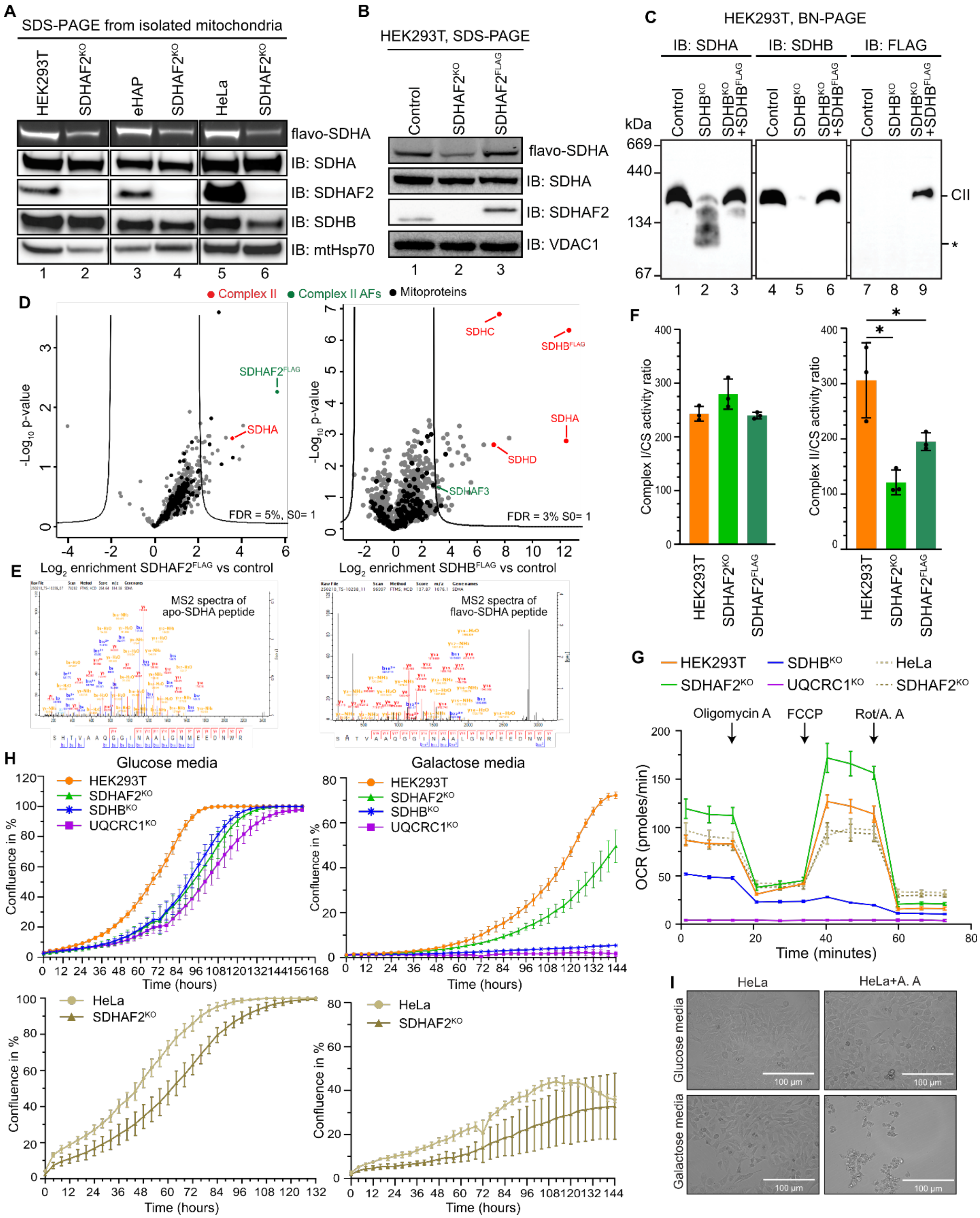
A
SDS-PAGE from isolated mitochondria
HEK293T
SDHAF2KO
eHAP
SDHAF2KO
HeLa
SDHAF2KO
flavo-SDHA
IB: SDHA
IB: SDHAF2
IB: SDHB
IB: mtHsp70
1 2 3 4 5 6
B
HEK293T, SDS-PAGE
Control
SDHAF2KO
SDHAF2FLAG
flavo-SDHA
IB: SDHA
IB: SDHAF2
IB: VDAC1
1 2 3
C
HEK293T, BN-PAGE
IB: SDHA
IB: SDHB
IB: FLAG
Control
SDHBKO
SDHBKO +SDHBFLAG
kDa
669
440
134
67
CII
*
1 2 3 4 5 6 7 8 9
D
Complex II
Complex II AFs
Mitoproteins
-Log10 p-value
SDHAF2FLAG
SDHA
FDR = 5%, S0= 1
Log2 enrichment SDHAF2FLAG vs control
SDHC
SDHBFLAG
SDHA
SDHD
SDHAF3
FDR = 3% S0= 1
Log2 enrichment SDHBFLAG vs control
E
MS2 spectra of apo-SDHA peptide
MS2 spectra of flavo-SDHA peptide
F
Complex I/CS activity ratio
Complex II/CS activity ratio
HEK293T
SDHAF2KO
SDHAF2FLAG
*
*
G
HEK293T
SDHBKO
HeLa
SDHAF2KO
UQCRC1KO
SDHAF2KO
Oligomycin A
FCCP
Rot/A. A
OCR (pmoles/min)
Time (minutes)
H
Glucose media
Galactose media
HEK293T
SDHAF2KO
SDHBKO
UQCRC1KO
Confluence in %
Time (hours)
HeLa
SDHAF2KO
I
HeLa
HeLa+A. A
Glucose media
Galactose media
100 µm

Fig. S1. Flavination of SDHA is required for the maturation of Complex II and is enhanced by SDHAF2. (**A**) Mitochondrial extracts were subjected to SDS-PAGE. Flavination of SDHA (flavo-SDHA) was detected in-gel through excitation at 488 nm (upper panels) and protein abundances determined by immunoblotting with indicated antibodies (lower panels). (**B**) As for A but with the indicated cell lines. (**C**) BN-PAGE analysis of Complex II assembly in HEK293T SDHB^{KO} and HEK293T SDHAB^{KO} + SDHB^{FLAG} cell lines, followed by immunoblotting with the indicated antibodies as described in Fig. 1A. (**D**) Whole cell lysates from HEK239T control, SDHAF2^{KO} + SDHAF2^{FLAG} and SDHB^{KO} + SDHB^{FLAG} cells were subjected to FLAG-immunoprecipitation mass-spectrometry analysis (IPMS). The curved line indicates significance determined through an FDR-based approach (FDR<5% or <3%, S0=1), with FLAG-enriched proteins on the right. n=3 technical replicates. (**E**) Representative MS/MS spectra of the SDHA peptide, with and without FAD-modification at His99. (**F**) Enzymatic activity of Complex I and II was measured in enriched mitochondrial fractions from HEK293T SDHAF2^{KO} + SDHAF2^{FLAG} cell line as described in Fig. 1F. Mean ± SD, n=3 technical replicates. *, $P < 0.05$. P-values were calculated using a one-way ANOVA. (**G**) Oxygen consumption rate (OCR) of the indicated HEK293T (solid lines) and HeLa (dotted lines) cell lines following sequential additions of oligomycin, FCCP, and rotenone plus Antimycin A, as indicated. Vertical bars indicate mean ± SD, n=5 technical replicates. (**H**) Growth comparison of control and SDHAF2^{KO} HeLa cells cultured in media containing glucose or galactose, monitored using an Incucyte Live cell imaging system. Vertical bars indicate mean ± SD, n=4 technical replicates. (**I**) Bright-field images taken 8 hours after treatment of HeLa cells with and without Antimycin A and cultured in glucose- or galactose-containing media, as indicated.

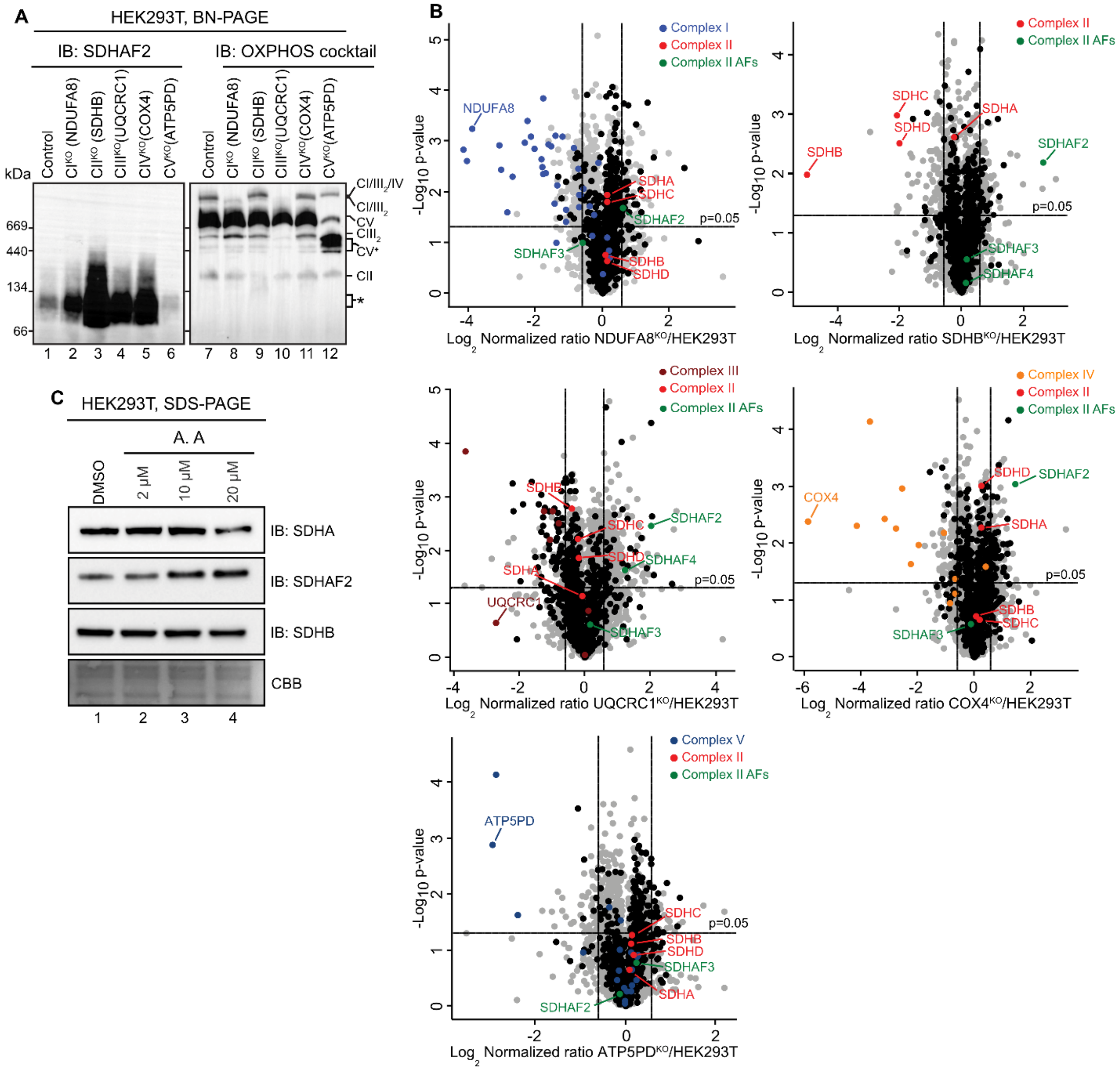

Fig. S2. The SDHAF2-SDHA sub-assembly accumulates during defective OXPHOS in HEK293T cells. (**A**) BN-PAGE analysis of control HEK293T, NDUFA8^{KO} (CI), SDHB^{KO} (CII), UQCRC1^{KO} (CIII), COX4^{KO} (CIV) and ATP5PD^{KO} (CV) mitochondria. Isolated mitochondria were solubilized in 1% digitonin and separated by BN-PAGE. Immunoblotting was performed using an antibody cocktail containing NDUFB8 (CI), SDHB (CII), UQCRC2 (CIII), MTCO1 (CIV) and ATP5A (CV). "*" denotes a subassembly of CII and "CV^{+}" denotes a subassembly of CV. (**B**) SILAC ratios for cellular proteins detected in the indicated control and knockout HEK293T cells. Related to Figure. 2A. Colored dots represent subunits or assembly factors of each OXPHOS complex as indicated. The horizontal line indicates P = 0.05 and the vertical line a fold change of 1.5. P-values were calculated using a two-tailed single sample Student's t-test. (**C**) SDS-PAGE analysis of SDHAF2 levels in HEK293T cells treated with the indicated concentrations of Antimycin A for 8 hours.

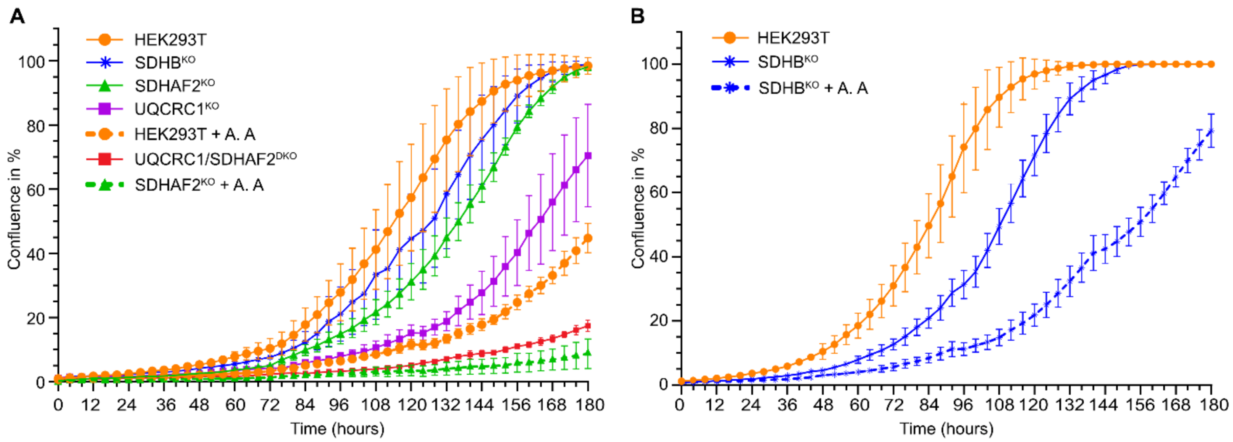

Fig. S3. Loss of SDHAF2 but not SDHB compounded with Complex III dysfunction leads to a severe growth defect in HEK293T cells. (A, B) Cellular proliferation of the indicated HEK293T cell lines, treated with or without Antimycin A, monitored using an Incucyte Live cell imaging system. Vertical bars indicate mean ± SD, n=4 technical replicates.

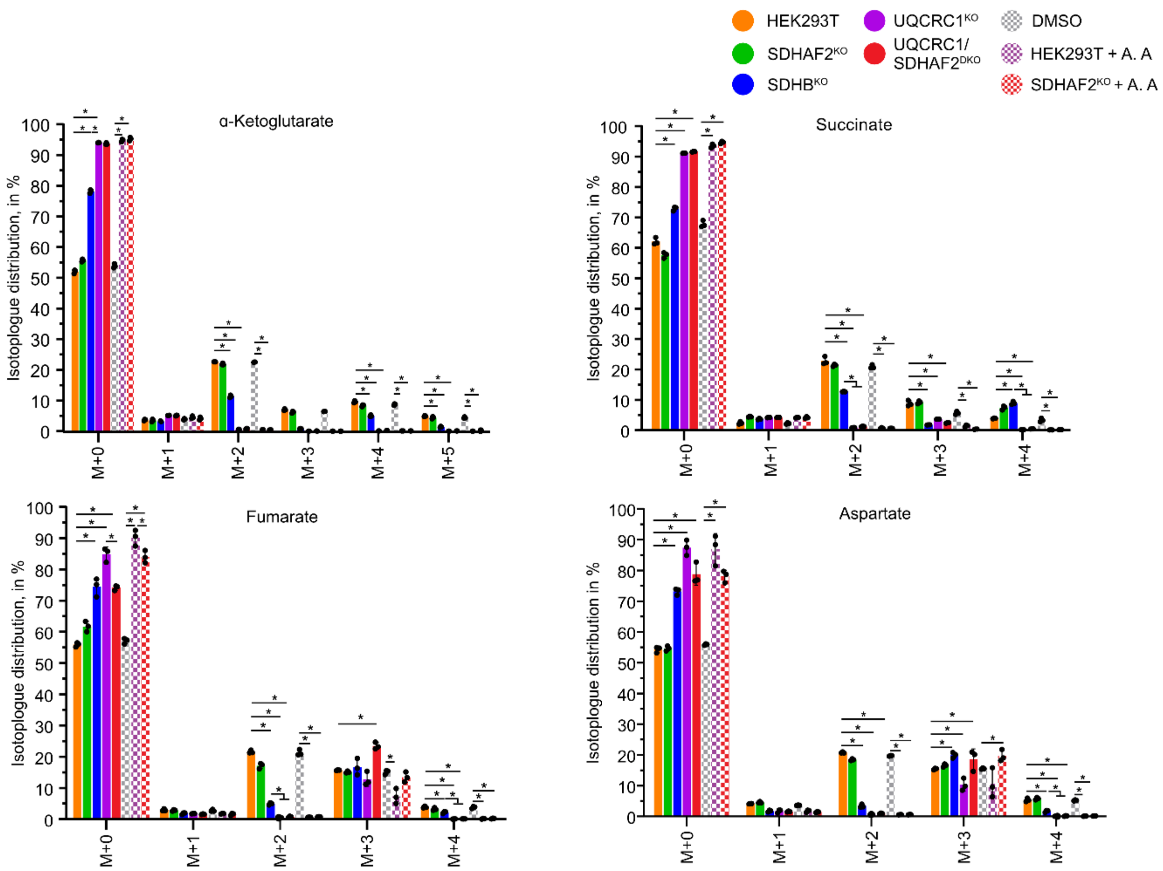

Fig. S4. Inhibition of Complex III compounded with loss of SDHAF2 leads to altered carbon utilization in HEK293T cells. Mass isotopologue distribution of α-ketoglutarate, succinate, fumarate and aspartate during $^{13}C_6$-glucose tracing in the indicated cell lines as per Fig. 4B. All data presented as mean ± SD following natural abundance corrections, n=3 biological replicates. *, P > 0.05. P-values were calculated using a one-way ANOVA.

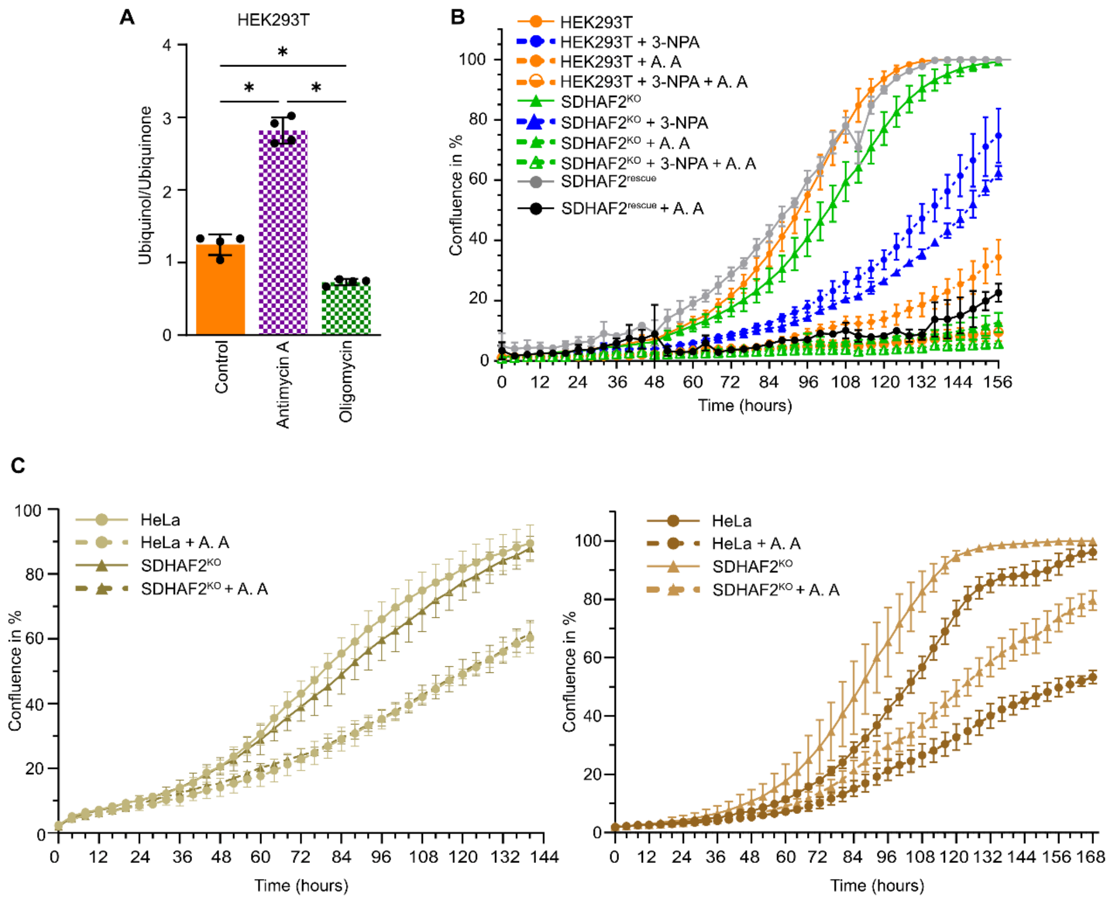

Fig. S5. **Inhibition of Complex III compounded with loss of SDHAF2 leads to cell-type specific growth phenotypes.** (**A**) Measurement of the ubiquinol/ubiquinone ratio in HEK293T cells treated individually with Antimycin A or Oligomycin for 8 hours, as described in the Materials and Methods. Mean ± SD, n=4 replicates. *, $P < 0.05$. P-values were calculated using a one-way ANOVA. (**B**) Growth analysis of the indicated HEK293T cells treated with either Complex II or Complex III inhibitors, or co-treated with both, monitored using the Incucyte Live-Cell Imaging System. Vertical bars indicate mean ± SD, n=4 technical replicates. (**C**) Growth analysis of HeLa and eHAP cells with and without Antimycin A treatment, performed as described in B.

Data S1. (separate file)

SILAC ratio data from a panel of OXPHOS knockouts generated in the HEK239T cell line. Individual Log2 transformed SILAC ratio (KO/WT), P-values from a single sample two-sided Student's t-test, and unique peptide counts for each protein were measured in the indicated cell lines.

Data S2. (separate file)

Migration profiles of SILAC-labelled mitochondrial proteins. Proteins were hierarchically clustered using NOVA, listed in MitoCarta 3.0, shown as heatmaps generated from iBAQ values with a color gradient indicating the min/max for each row (first tab). Entries are curated according to OXPHOS subunits, with all remaining mitochondrial entries identified in the experiment ordered according to hierarchical clustering. For downstream heatmap analysis, individual iBAQ values were normalized by the average iBAQ intensities in control HEK293T cells for their respective protein. For profile plots, individual iBAQ values were normalized by the maximum intensity of the proteins being compared. Complexome mass calibration, using selected protein apparent masses, is shown in the second tab.

Data S3. (separate file)

Steady state levels of polar metabolites in HEK293T SDHB^{KO} and UQCRC1^{KO} compared to WT cells. Individual Log2 transformed values were normalized by subtracting the median of each column, followed by a single-sample Student's T-test analysis.
